# Short Total Synthesis of Ajoene, (*E*,*Z*)‐4,5,9‐Trithiadodeca‐1,6,11‐triene 9‐oxide, in Batch and (*E*,*Z*)‐4,5,9‐Trithiadodeca‐1,7,11‐triene in Continuous Flow

**DOI:** 10.1002/chem.202001598

**Published:** 2020-06-15

**Authors:** Marina Yamamoto Raynbird, Filipa Silva, Harry Smallman, Shaista S. Khokhar, Daniel Neef, Gareth J. S. Evans, Thomas Wirth

**Affiliations:** ^1^ School of Chemistry Cardiff University Park Place, Main Building Cardiff CF10 3AT UK; ^2^ Neem Biotech Roseheyworth Business Park North Abertillery NP13 1SX UK

**Keywords:** ajoene, disulfides, flow chemistry, garlic, organosulfur compounds

## Abstract

A short total synthesis of ajoene, (*E,Z*)‐4,5,9‐trithiadodeca‐1,6,11‐triene 9‐oxide, has been achieved over six steps. In addition, a continuous flow synthesis under mild reaction conditions to (*E,Z*)‐4,5,9‐trithiadodeca‐1,7,11‐triene is described starting from simple and easily accessible starting materials. Over four steps including propargylation, radical addition of thioacetate, deprotection, and disulfide formation/ allylation, the target product can be obtained at a rate of 0.26 g h^−1^ in an overall yield of 12 %.

## Graphical abstract

**Get into the flow**! A continuous flow synthesis of a novel sulfide is reported over four steps and has been biologically evaluated. In addition, a novel synthesis of ajoene has been established (see figure).

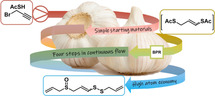

Garlic extracts are frequently reported in the literature for exhibiting a wide range of biological properties. For example, ajoene (**3**), a component that is derived from naturally occurring compounds in garlic, is a potent antithrombotic agent with platelet aggregation inhibitory properties.^1^ Other compounds obtained from garlic extracts have shown to be beneficial against coronary thrombosis and atherosclerosis, as well as displaying a range of antibacterial and antifungal activities.^2^, ^3^ (*E*/*Z*)‐Ajoene **3** was first observed in 1983 by Apitz‐Castro,^4^ however, it was not until 1984 that **3** was fully characterised by E. Block.^1^ Block and co‐workers have obtained (*E*/*Z*)‐**3** through a thermal rearrangement of allicin (**2**), which itself is formed from alliin **1** (Schemes 1 a,b). Although Block isolated **3** in 37 % yield as a mixture of its geometric isomers, an array of other rearrangement and decomposition products were also obtained, including mono and polysulfides as well as vinyl‐dithiins.

**Scheme 1:**
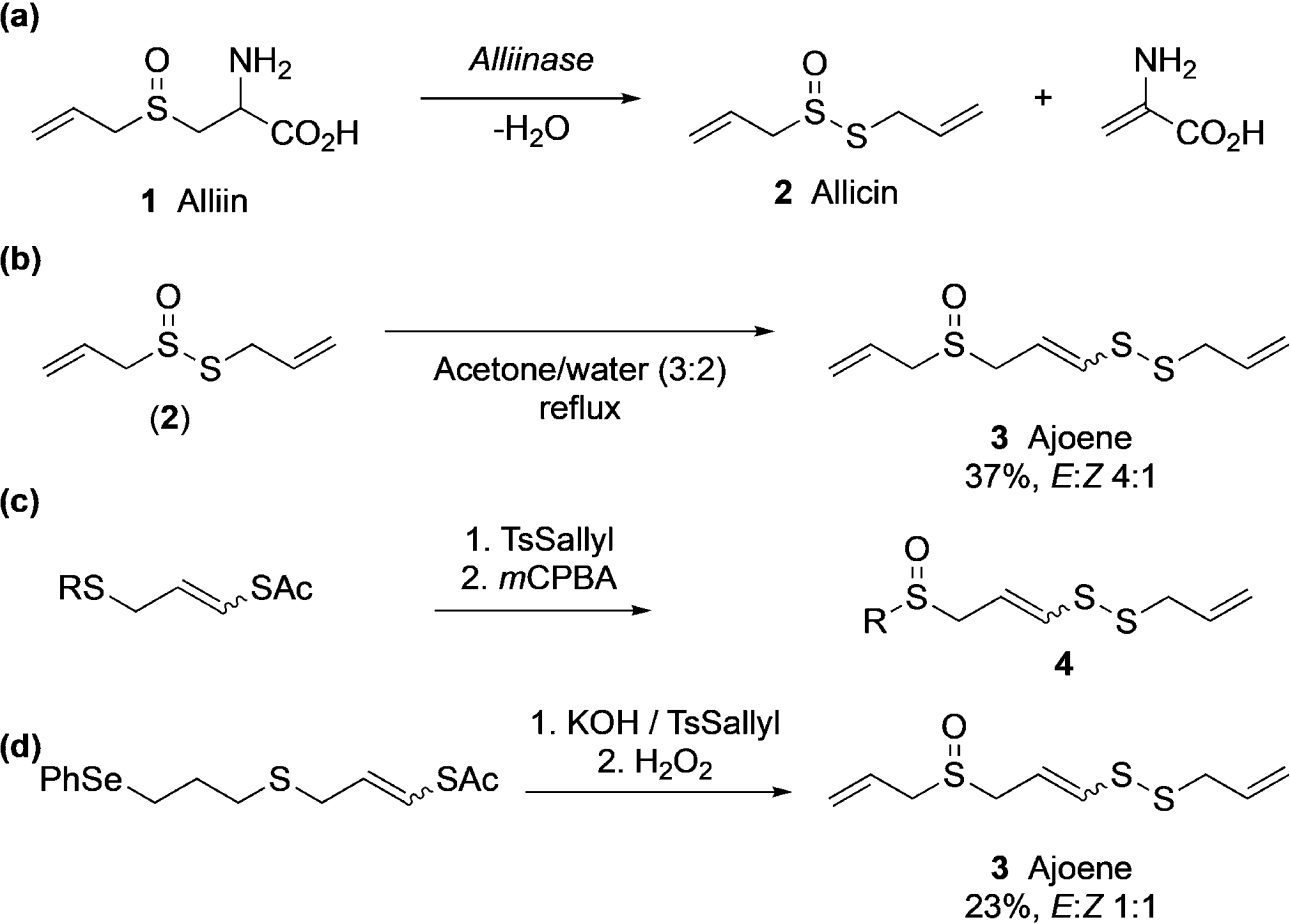
Synthesis of ajoene **3** as described by Block et al. in 1984. (a) Formation of allicin **2** from alliin **1** by the action of alliinase. (b) Formation of *E*/*Z*‐ajoene **3** from **2** by thermal rearrangement in aqueous acetone. (c) Synthesis of ajoene analogues **4** by Hunter et al. (d) Synthesis of ajoene **3** by Silva et al.^10^

Since 1984 there have been numerous reports of oxidising diallyl disulfide using simple oxidants such as *meta*‐chloroperbenzoic acid,^5^ Oxone®^6^ and hydrogen peroxide,^7^ thus eliminating the requirement for an enzymatic process.

Analogues of **3** have been reported in the literature in the search of synthetic analogues **4** with higher bioactivity than **3**. Hunter et al. described a synthetic route for the synthesis of substituted ajoene analogues **4** where the terminal group of the sulfoxide is varied (7 examples, Scheme 1 c). However, an allyl group at the sulfoxide, thus yielding **3** as final product, could not be obtained due to the instability of the vinyl radical precursor that would be required.^8^ The scope of the analogues were extended later when both terminal allyl moieties of the ajoene have been altered.^9^

In 2018, we reported the first total synthesis of **3** (Scheme 1 d). The chemical total synthesis overcame such obstacles such as the instability and volatility of **2** by replacing allicin with alternative starting materials to develop a reliable and robust five step synthesis.^10^

We were able to install the terminal allyl group from the sulfoxide using a selenoxide elimination as key step. Using two equivalents of a simple oxidant such as hydrogen peroxide, oxidation of the sulfur to sulfoxide occurred as well as the selenide to the selenoxide. Due to the presence of β‐hydrogens, the selenoxide spontaneously eliminates furnishing the terminal allyl functionality. Despite this novel route overcoming some previous obstacles, the total synthesis has inherent drawbacks. These are, firstly, the difficulty of scaling‐up due to the use of selenium, and secondly, a low overall yield.

Herein, we describe a novel synthesis of sulfide **8** where four steps have been performed in continuous flow with high atom economy. Originally intended to be a precursor for the synthesis of ajoene **3**, it was found that **8** is an isomeric sulfide leading to a revised short total synthesis of **3**. The new route eliminates the need for selenium or high molecular weight protecting groups.

In order to install the terminal allyl group, the synthetic route herein utilises thioacetic acid rather than a free thiol (Scheme 2). The acetate protecting group provides stability to the resulting vinyl radical intermediate that could form under the conditions of radical formation with AIBN. An additional advantage to being a facile group to cleave, is that the acetate will provide acetic acid as the only by‐product.

**Scheme 2:**
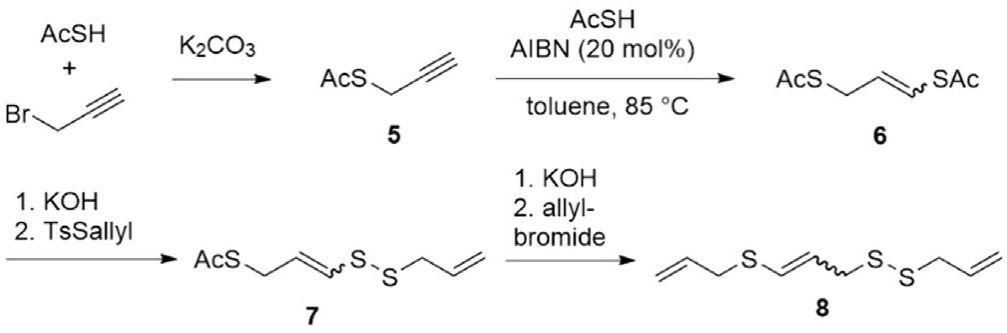
Short synthesis of potential isomeric ajoene precursor **8**.

The batch propargylation of thioacetic acid with organic bases such as triethylamine and diisopropyl ethylamine were unsuccessful. It was found that potassium carbonate in a mixed solvent system of THF/CH_2_Cl_2_ (1:1) yielded **5** in up to 95 % yield after 4 hours at room temperature while purification was not required. Solvents such as toluene and CH_2_Cl_2_ were also investigated although required longer reaction times. Using toluene as solvent complicated isolation of **5** due to the volatility of the product being higher than the solvent.

In the development of the corresponding flow process, an OmniSep® column was packed with a mixture of potassium carbonate and sand. This mixture reduced the build‐up of pressure as the base is consumed over time and allowed the flow rate to stay consistent. When using a mixed solvent system, the eluted mixture showed that the reaction had not achieved complete conversion (Table 1, entries 1–5) despite an increased loading of base or an increased residence time. In contrast, where THF was employed as solvent, the reaction was able to achieve complete conversion (Table 1, entries 7–10) where **5** was obtained in yields of up to 84 % in 18 minutes. Increasing the base percentage to 56 wt.% saw a decrease in yield, likely to be the result of the product being unable to elute efficiently from the column. Entry 10 confirmed the reproducibility of entry 8 where a larger column, and therefore an increased residence time, was used.

**Table 1:** Propargylation of thioacetic acid to obtain thioester **5** in flow.^[a]^

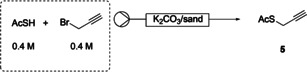				
Entry	Solvent	Base [wt %]	Residence *t* [min]	Yield **5** [%]
1	THF/CH_2_Cl_2_ (1:1)	7	20	19
2	THF/CH_2_Cl_2_ (1:1)	7	40	42
3	THF/CH_2_Cl_2_ (1:1)	7	60	45
4	THF/CH_2_Cl_2_ (1:1)	14	30	49
5	THF/CH_2_Cl_2_ (1:1)	21	30	46
6	THF	7	20	50
7	THF	21	30	79
8	THF	42	18	84
9	THF	56	14	57
10	THF	42^[b]^	24	82

[a] Conditions: HPLC K120 Knauer Analytical pump with thioacetic acid and propargyl bromide (0.4 m) in dry solvent, column: K_2_CO_3_/sand (20 g total) in OmniSep® column (15×150 mm). [b] Column: repacked Biotage® SNAP Ultra cartridge (30 g total). The column volume and, therefore, flow rate was varied between each run due to the percentage of base.

For the generation of the central double bond, a radical addition of thioacetate to **5** was employed using azobisisobutyronitrile (AIBN) as initiator. A range of solvents were investigated in batch where toluene gave the largest isolated yield of **5**, 60 % (Scheme 3). Since the continuous in‐flow system would require each step to be compatible with the previous step, tetrahydrofuran and dichloromethane were also investigated. The presence of dichloromethane seemed to be advantageous and increased the yield from 8 % to 51 % when in a ratio of (1:3) with tetrahydrofuran.

**Scheme 3:**
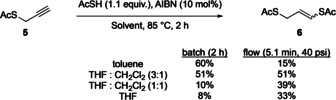
Batch reaction for the radical addition of thioacetic acid to terminal alkyne **5**.

Translating the batch reaction to a flow system required the use of a Uniqsis® Heated Reactor to heat the PTFE reactor coil to 85 °C. A back‐pressure regular (40 psi) was fitted to ensure that the system remained homogenous, despite the evolution of nitrogen gas. A mixed solvent system of THF:CH_2_Cl_2_ (3:1) gave the highest yield of **5** in flow and was isolated in 51 % yield (Scheme 3).

Since the solvent system of first two steps were similar, both steps could be coupled easily, and the resulting flow system was used for the investigation of the residence time in step 2. In order to achieve a THF:CH_2_Cl_2_ (3:1) solvent mixture in step 2, step 1 was performed in THF and AIBN and thioacetic acid was added in a solution of THF:CH_2_Cl_2_ (1:1). Column 2 (C2) was trialled with PTFE tubing of a reduced internal diameter (0.2 mm). However, this gave inconsistent results because of varying flow rates due to increased pressure. Therefore, a PTFE tubing with internal diameter of 0.8 mm was used. The increase in residence time from 6 minutes to 38 minutes for C2 resulted in a yield of **6** of 61 % over two steps (Scheme 4). Longer residence times for C2 were not investigated due to practical considerations as the time would exceed one hour for the first two steps. Boron‐based radical initiators were also considered, although these gave a less clean reaction with inferior yields of **6**.

**Scheme 4:**
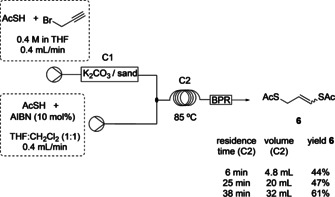
Investigating the effect of residence time on the yield of product **6** over two coupled flow steps.

The subsequent deprotection of the vinylic sulfur atom proceeded cleanly using potassium hydroxide in methanol with good selectivity for the vinylic thioacetate, however, the other thioacetate was also cleaved partially and side product **8** was formed (Table 2). The concentration, temperature and residence time, along with equivalents of the allyl thiotosylate electrophile and potassium hydroxide were investigated and results are summarised in Table 2. Entries 1 and 2 indicate that a more concentrated reaction leads to a less selective cleavage of the acetate group. The temperature was reduced from room temperature (entry 3), resulting in an increase in yield of **7** to 46 %. Despite varying the residence time, concentration and temperature further (entries 4–5), no significant differences in the isolated yields of **7** or **9** were observed. To increase the yield of desired product, a Design of Experiments (DoE) study was conducted to indicate a reaction ‘sweet‐spot’ (see supporting information). In this study, residence time, temperature and the equivalents of electrophile and base were investigated. The optimum reaction conditions as a result of this DoE study are given in entry 7, where the equivalents of allyl thiotosylate and base were increased to obtain **7** in 56 % yield. This result was also repeated with a mixed solvent system of THF:CH_2_Cl_2_:MeOH (3:1:4) giving a similar result. The comparable batch reaction for the deprotection and disulfide formation yields **7** in 51 % and **9** in 10 %, similar to recent publications on the synthesis of disulfides from thioacetates and thiosulfonates.^11^

**Table 2:** Flow set‐up for the investigation of step 3 to yield **7**.^[a]^

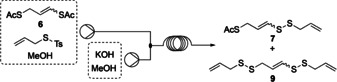					
Entry	*c* **6** [m]	*T* [°C]	Residence *t* [min]	Yield **7** [%]	Yield **9** [%]
1	0.1	r.t.	5	35	9
2	0.2	r.t.	5	22	10
3^[b]^	0.1	0	5	46	9
4^[b]^	0.1	0	10	41	6
5^[b,c]^	0.1	−40	10	34	31
6^[a]^	0.05	−5 to −10	15	46	11
7^[b,d]^	0.1	0	5	56	10

[a] Reagents and conditions: Vinyl thioacetate **6** (1.0 equiv), allyl thiotosylate (1.1 equiv) in MeOH, KOH (1.05 equiv.) in MeOH. C.V.: 1 mL, id: 0.8 mm. [a] Coil submerged in cold bath. [c] Reagents and conditions: KOH (1.4 equiv). [d] Reagents and conditions: allyl thiotosylate (3 equiv), KOH (2 equiv).

Bisdisulfide compound **9** is a natural product that has been extracted from Chinese garlic with acetone.^12^ The flow system therefore provides the first reported chemical synthesis of **9**. Entry 5 shows the potential to tune the reaction toward the bisdisulfide **9**. Where the equivalents of base are greater than that of the electrophile, the reaction favours the formation of **9**. Increasing the equivalents further would increase yields of **9** to give a selective reaction.

With the vinylic disulfide moiety installed in **7**, the deprotection of the second acetate was investigated in flow using allyl bromide as electrophile. The equivalents of allyl bromide and base were controlled through the concentration whilst keeping the flow rate constant. The effect of temperature using caesium carbonate as base and the equivalents of allyl bromide were varied and saw an improvement in yield from 12 % to 20 % by increasing the base concentration. Potassium hydroxide gave an increased yield of **8** to 29 % under the same conditions as caesium hydroxide. To optimise the yield of product **8**, a DoE study was conducted (see supporting information). The optimum reaction conditions as a result of the DoE study indicate that the equivalents of allyl bromide and potassium hydroxide should be increased to 3 to obtain **8** in 43 % yield. Sodium hydroxide, ammonium hydroxide and caesium carbonate as bases were also investigated, although these gave inferior yields. The comparable batch reaction using allyl bromide and caesium carbonate for the deprotection and allylation formation yields **8** in 45 % yield (Scheme 5).

**Scheme 5:**
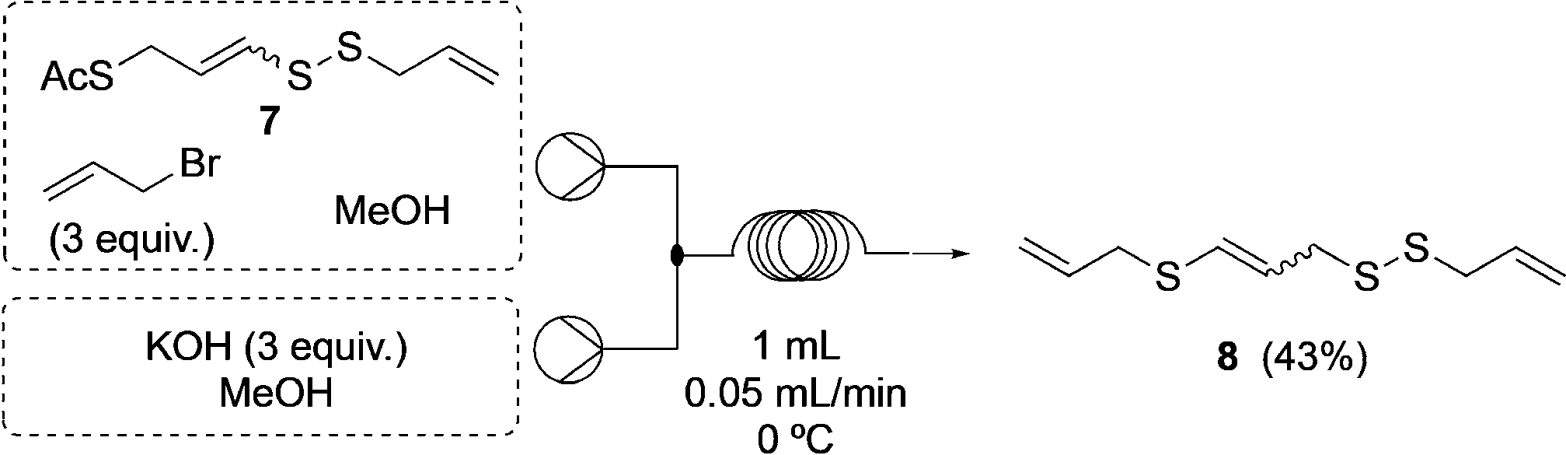
Flow setup for step 4 to yield compound **8**.

With each step optimised, all four steps were combined, and the resulting system was tested. Calculating from the yields of the individual steps (1 and 2: 61 %; 3: 56 %; 4: 43 %), an overall yield of 15 % was expected. To our delight, **8** could be obtained in 12 % as the major product with a production rate of 0.26 g h^−1^ (Scheme 6). This is comparable to the batch reactions where **8** can be obtained in 13 % yield. Although the flow synthesis does not currently improve upon the batch yield, it provides the first example of a continuous flow synthesis for the potential precursor of ajoene **3** without any inline purification. This saves time, energy and resources as only a single silica gel chromatography column is required in the purification of **8** after the reaction sequence.

**Scheme 6:**
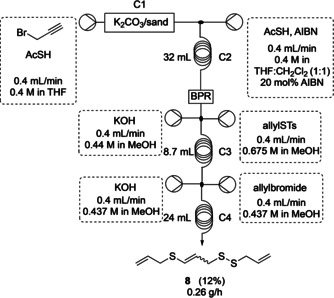
Flow setup for all consecutive reactions to produce compound **8**.

However, it was not compound **8** but **12** which was intended to be the product of the flow synthesis. It turned out that, after deprotection of the acetate in **7**, the resulting thiolate **10** reacts with the disulfide moiety in an intra‐ or intermolecular fashion as this is a reactivity well known also in biological systems.^13^ The product is the more stable vinyl thiolate **11** which is being allylated to give **8**. While oxidation of **12** would lead to ajoene **3**, oxidation of **8** with *m*CPBA resulted in the formation of aldehyde **13** (Scheme 7).^14^ This transformation is suspected to be the result of epoxidation of the central olefin, followed by migration of the allyl sulfide with ring opening of the epoxide to form the aldehyde. Alternatively, treatment of **8** with *m*CPBA could form the sulfoxide, which in turn could undergo a thio‐Claisen rearrangement.^15^

**Scheme 7:**
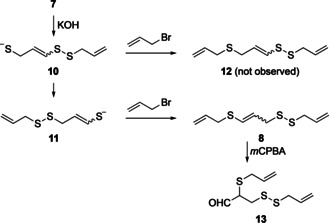
Proposed mechanism for the formation of compound **8**.

Compound **8**, as the major product of the flow system, and compound **9**, a natural product obtained as a side product of the flow system, were biologically evaluated by Neem Biotech Ltd. and compared with naturally obtained ajoene. Biological evaluation results are summarised in Table 3. Compounds were tested against *Staphylococcus aureus* (*S. aureus*) and *Pseudomonas aeruginosa* (*P. aeruginosa)*, bacteria which are commonly associated with chronic wounds. The assays conducted were Minimum Biofilm Inhibition Concentration (MBIC) and Quorum Sensing Inhibition (QSI) studies. The former determines the minimum concentration of a compound required to limit the biofilm production to 50 %, when compared with bacteria in the absence of the compound. The results for *S. aureus* MBIC assay were comparable between entries 1–3, however, a larger difference could be observed against *P. aeruginosa*. In this case, compound **8** showed to be significantly more active resulting in an IC_50_ value 37 % lower than ajoene and 58 % lower than its mono‐disulfide analogue. This suggests that the disulfide moiety in the compound plays a significant role its mode of action. Interestingly, compound **8** displayed no activity in QSI studies against *S. aureus*.

**Table 3:** Biological evaluation of ajoene, **8** and **9**.

Entry	Compd.	*S. aureus* MBIC IC_50_ [μm]	*P. aeruginosa* MBIC IC_50_ [μm]	*P. aeruginosa* QSI [% at 75 μm]	*S. aureus* QSI [mm]
1	ajoene **3**	0.69	45	78	30
2	**8**	<2.25	67.5	94	35
3	**9**	<2.25	28	89	0

In light of the above findings, a revised synthesis to ajoene **3** has been developed to overcome the scrambling of the disulfide and sulfide positions. The radical addition of 4‐methoxybenzyl thiol affords product **14** with both an acid and base sensitive protecting group on both sulfur atoms. The unambiguous deprotection of the acetate allows for allylation at the desired sulfur atom yielding **15** in 88 % yield. Treatment under acidic conditions deprotects the *para*‐methoxy benzyl (PMB) group and traps the resulting alkenethiol as the thioacetate **16**. This occurs in situ as the alkenethiol is a strong nucleophile and reacts with the activated acetic acid. Trapping the thiol as **16** allows to employ the basic reaction conditions needed for the disulfide formation. Treating a PMB protected thiol with trifluoroacetic anhydride (TFAA), and then directly treating with KOH and TsSallyl were trialled in a previous project but did not result in the desired product. The yield suffers due to the instability and volatility of the deprotected alkene thiol intermediate. Other thiol protecting groups have not been considered here. With potassium hydroxide and allyl thiotosylate formation of the disulfide bond in the vinylic position is achieved in good yield (70 %). Finally, oxidation of sulfide **12** affords ajoene **3** in 49 % yield (Scheme 8).

**Scheme 8:**
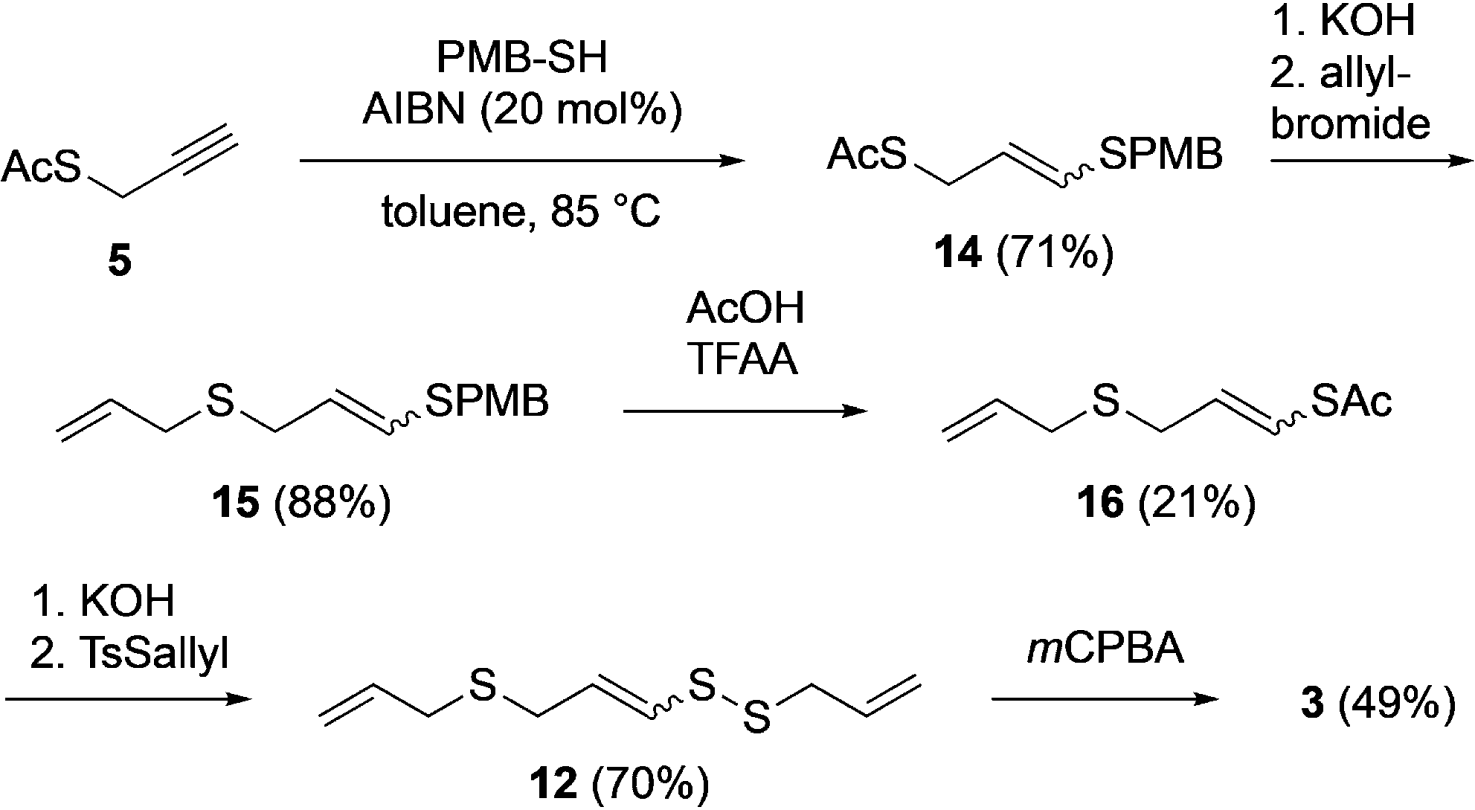
Batch synthesis of ajoene **3** over 6 steps.

In conclusion, a short, novel synthesis of sulfide **8** has been established in batch and a subsequent continuous flow system has been developed. In addition to this, a revised short synthesis of ajoene **3** has also been established using two different protecting groups. Biological evaluations of sulfide **8** and the natural product **9** have been conducted in comparison to ajoene **3** which show promising results for quorum sensing inhibition in *P. aeruginosa*.

## Experimental Section

### Flow procedure for the synthesis of S‐(prop‐2‐yn‐1‐yl) ethanethioate 5

Potassium carbonate (8.4 g, 0.06 mol) and sand (11.6 g) were shaken until a homogenous mixture was achieved. The K_2_CO_3_/sand (20 g) was packed into a glass OmniSep® (150×15 mm). The packed column was weighed, and dry tetrahydrofuran (THF) was flowed through at 0.4 mL min^−1^ for 1 hour, after which, the column was reweighed. The mass difference equated to the calculated column volume (CV, 7.1 mL, residence time: 18 minutes). To an oven dried glass, thioacetic acid (0.4 m) and propargyl bromide (0.4 m) were added to dry THF under argon. The reaction mixture was passed through the column at 0.4 mL min^−1^ using a syringe pump and the first column volume of solution was discarded. The following two column volumes were collected and concentrated in vacuo at room temperature to yield product **5** as a yellow oil (453 mg, 95 %).

## Conflict of interest

The authors declare no conflict of interest.

## Supporting information

As a service to our authors and readers, this journal provides supporting information supplied by the authors. Such materials are peer reviewed and may be re‐organized for online delivery, but are not copy‐edited or typeset. Technical support issues arising from supporting information (other than missing files) should be addressed to the authors.

Supplementary
