# Supplementary material for: Short Total Synthesis of Ajoene, (E,Z)‐4,5,9‐Trithiadodeca‐1,6,11‐triene 9‐oxide, in Batch and (E,Z)‐4,5,9‐Trithiadodeca‐1,7,11‐triene in Continuous Flow
Source: Chemistry. 2020 Jun 15;26(38):8363–7. doi: 10.1002/chem.202001598 (PMC7384054; doi:10.1002/chem.202001598)
Supplement: Supplementary file 1 — Supplementary [file CHEM-26-8363-s001.pdf]

Chemistry–A European Journal

Supporting Information

Short Total Synthesis of Ajoene, (*E*,*Z*)-4,5,9-Trithiadodeca-1,6,11-triene 9-oxide, in Batch and (*E*,*Z*)-4,5,9-Trithiadodeca-1,7,11-triene in Continuous Flow

Marina Yamamoto Raynbird,[a] Filipa Silva,[a] Harry Smallman,[a] Shaista S. Khokhar,[b] Daniel Neef,[b] Gareth J. S. Evans,[b] and Thomas Wirth*[a]

Supporting Information

General Considerations

The reactions were performed using standard laboratory equipment. Air sensitive reactions were carried out under argon atmosphere using oven-dried glassware. Reactions were stirred using magnetic stirring and heated to specified temperatures using hotplates with temperature probe control and an adapted heating block. Lower temperatures were obtained using ice/water (0 ºC) and dry ice/acetonitrile (–40 ºC). Büchi B-461, B-481 or B-490 were used for solvent evaporations (reduced pressure up to 15 mbar) and high vacuum apparatus was used to further dry the products. All chemicals were purchased from Sigma Aldrich, Alfa Aesar, Fisher Scientific, TCI UK, Fluorochem and used without further purification. Dry solvents were obtained from an MBRAUN SPS-800 solvent purification system.

All the reactions were monitored by thin-layer chromatography (TLC), which was performed on Merck Silica gel 60 F254 (0.20 m) and visualised by UV radiation (254 nm) or/and by staining with potassium permanganate solution (1.5 g $KMnO_4$, 10 g K_2CO_3, 1.25 mL 10% NaOH, 200 mL distilled H_2O). Automated column chromatography was performed on a Biotage® Isolera Four using Biotage® cartridges SNAP Ultra 10 g, SNAP Ultra 25 g, SNAP Ultra 50 g, SNAP Ultra 100 g. The solvents used for the purification are indicated in the text and were purchased from Fisher Scientific as laboratory grade.

^{1}H NMR and ^{13}C NMR spectra were measured on Burker DPX 500 (500 MHz), Bruker DPX 400 (400 MHz), Bruker DPX 300 (300 MHz) instruments. The chemical shifts: δ = are given in ppm downfield of tetramethylsilane (δ = 0 ppm). Compounds or crude reaction mixtures were dissolved in deuterated chloroform. Coupling constants (*J*) are given in Hertz. The multiplicity of signals is designated: s = singlet, d = doublet, t = triplet, q = quartet, quin= quintet, dt = double of triplet, m = multiplet. Residual solvent peaks are 7.26 ppm for chloroform. The molecular ions peaks values quoted for either molecular ion $[M]^+$, molecular ion plus hydrogen $[M+H]^+$, molecular ion plus sodium $[M+Na]^+$, molecular ion plus ammonium $[M+NH_4]^+$ or molecular ion plus $[M+K]^+$. IR spectra were recorded on Shimadzu IR Affinity-1S apparatus. Wavenumbers are quoted in cm^{-1}.

General Scheme 1

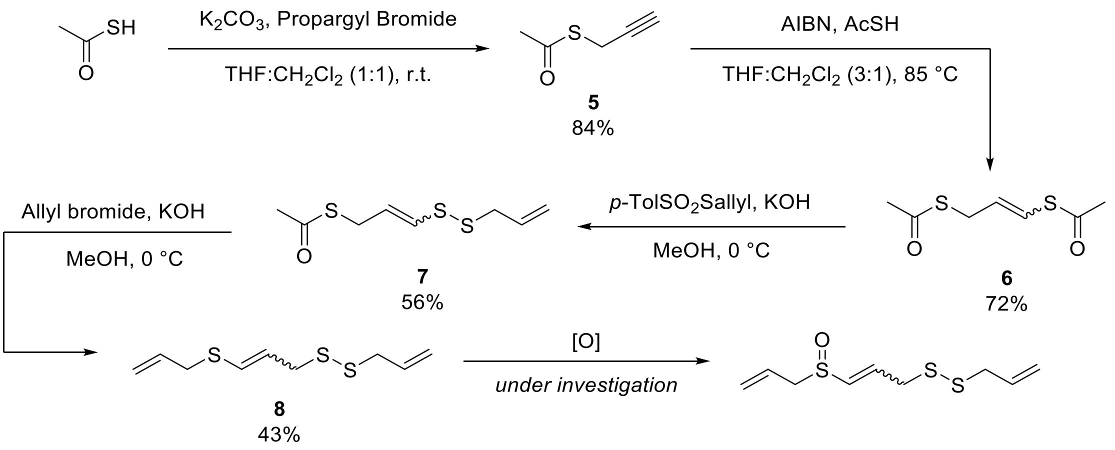

General Scheme 2

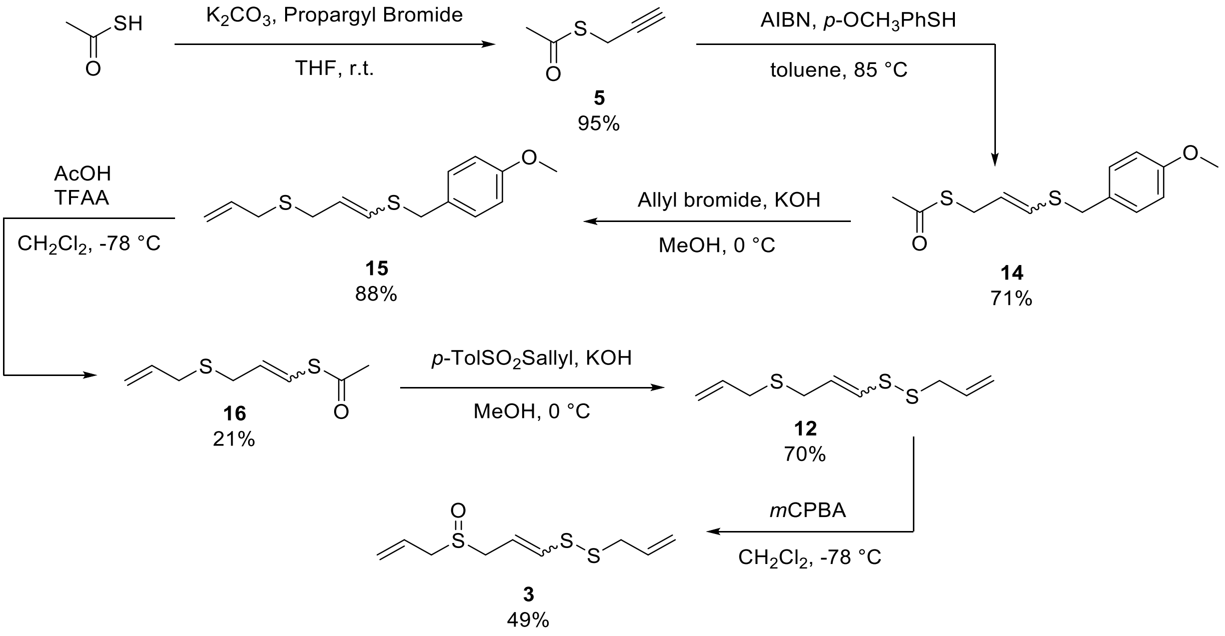

Biological Assay Techniques

1. MBIC Assay[1]

In short, compounds were assayed for their ability to inhibit S. aureus or P. aeruginosa biofilm formation as follows. All compounds were prepared as a 30 mM stock in DMSO and diluted in TSB to 144 µM. The compounds were further serially diluted two-fold from 144 µM to 2.25 µM and added to a 96-well plate at 100 µL per well. Overnight cultures of bacteria were diluted 1:2000 in fresh TSB and 100 µl of the diluted bacterial culture was added to the compound containing wells. The bacteria were incubated in the presence of the compounds overnight at 37 ºC. After incubation, the planktonic bacteria were removed by three washes using H_2O and the remaining biofilm was stained using crystal violet and quantified photometrically at A570. Absorbances corresponding to biofilm mass were plotted versus compound concentration and IC50 values were calculated using Graphpad Prism 8.0.

2. Pa QSI Assay[2]

To summarise, the P. aeruginosa lasB-gfp strain was cultured overnight in ABT with gentamicin (selective antibiotic; 120µg/mL) at 37 ºC, 150 rpm. Test compounds were solubilized to a stock concentration of 30 mM in DMSO (100%) and diluted 1:100 in ABT media. The compounds were further diluted 1:1 in the wells of a black 96 well plate to produce a range of test concentrations; the final concentrations tested were 150 µM, 75 µM, 37.5 µM and 18.75 µM. ABT medium without compound was added to rows one and twelve of the 96 well plate to provide untreated controls and blank medium controls respectively. The overnight culture was diluted to an OD600 of 0.08 in ABT medium (the values were blanked against ABT medium) and added 1:1 to each well with the exception of the blank medium control. The plate was placed into the BMG Fluostar Omega whereby the plate was read for absorbance (OD600) and fluorescence (excitation 485 nm, emission 520 nm) every 15 minutes for 16 hours at 37 ºC. The resulting readings were blank corrected, replicates were averaged, and data were normalized (Relative Fluorescence Units (RFU)/OD). The maximum fluorescence of each sample was compared to the mean maximum fluorescence of the untreated controls; the ratio was expressed as percent inhibition. Each compound was tested 3 times, except for ajoene which was tested 6 times. The data were reported as the percent inhibition of fluorescence when treated with 75 µM compound, a measure of the inhibition of expression of the lasB gene in P. *aeruginosa*.

3. Sa QSI Assay[3]

30 mM stock solutions of E/Z-ajoene and ajoene precursors of the General Scheme 1 and 2 were further diluted to a top concentration of 3 mM using 100% DMSO. The top concentration was then serially diluted 1:1 with DMSO to achieve ajoene/analogue concentrations ranging from 3 mM to 0.09375 mM. An overnight culture of spa:lacZ was diluted 1:1000 in fresh TSB and 800 µL was added to a flask containing 20 mL molten TSA, 5 µg/mL erythromycin and 150 µg/mL X-Gal. This mixture was poured into a sterile petri dish and allowed to solidify. Six blank antimicrobial discs (Oxoid) were added to the agar plate and labelled 1-6. 10 µL of diluted compound was added to each disc, with 1 representing the highest concentration (3 mM) and 6 representing the lowest concentration (0.09375

mM). The plates were incubated at 37 ºC for 16 h. Data were measured as the diameter (mm) of the zone of blue colouration around the disc.

Experimental Part

***S*-(Prop-2-yn-1-yl) ethanethioate (5)**

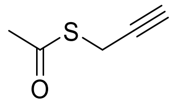

Batch Procedure:

To a solution of thioacetic acid (0.35 mL, 5 mmol) in THF (15 mL) were added propargyl bromide (0.43 mL, 5 mmol) and potassium carbonate (0.691 g, 5 mmol). The mixture was stirred at room temperature for 4 hours. The mixture was filtered, and the solid washed with dichloromethane (2 x 20 mL). The mixture was concentrated *in vacuo* at room temperature to yield **5** as a yellow oil (0.57 g, 95%).

Flow Procedure:

Potassium carbonate (8.4 g, 0.06 mol) and sand (11.6 g) were shaken until a homogenous mixture was achieved by eye. The K_2CO_3/sand (20 g) was packed into a glass OmniSep® (150 x 15 mm). The packed column was weighed, and dry tetrahydrofuran (THF) was flowed through at 0.4 mL/min for 1 hour, after which, the column was reweighed. The mass difference equated to the calculated column volume (CV, 7.1 mL, residence time: 18 minutes).

To an oven dried glass, thioacetic acid (0.4 M) and propargyl bromide (0.4 M) were added to dry THF under argon. The reaction mixture was passed through the column at 0.4 mL/min and the first column volume of reactant was discarded. The following two column volumes were collected and concentrated *in vacuo* at room temperature to yield **5** as a yellow oil (453 mg, 95%).

^{1}H NMR (300 MHz, $CDCl_3$): δ = 3.64 (d, *J* = 2.7 Hz, 2H, $CH_2C{\equiv}CH$), 2.37 (s, 3H, CH_3), 2.18 (t, *J* = 2.7 Hz, 1H $CH_2C{\equiv}CH$).
^{13}C NMR (75 MHz, $CDCl_3$): δ = 194.0 (C=O), 78.9 ($CH_2C{\equiv}CH$), 71.0 ($CH_2C{\equiv}CH$), 30.3 (CH_3), 17.6 ($CH_2C{\equiv}CH$).
Spectra in accordance with literature.[4]

***S*,*S*'-(Prop-1-ene-1,3-diyl) diethanethioate (6)**

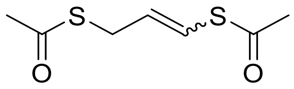

Batch Procedure:

S-(Prop-2-yn-1-yl) ethanethioate **5** (398 mg, 3.5 mmol) was dissolved in dry toluene (5 mL) and the solution was heated to 85 °C under argon atmosphere. Azobisisobutyronitrile (AIBN) (57 mg, 0.35 mmol, 0.1 equiv.) was added to the solution directly, followed by the dropwise addition of thioacetic acid (0.28 mL) in toluene (5 mL) over 40 minutes using a syringe pump. The mixture was left to stir at 85 °C for a further 1 hour. The reaction was then quenched with aqueous saturated solution of sodium carbonate

(5 mL) and the toluene was removed *in vacuo*. The remaining residue was dissolved diethyl ether (20 mL) and the organic layer was washed with brine (2 x 10 mL) and dried over $MgSO_4$. The solvent was evaporated *in vacuo* and the resulting residue was purified by column chromatography using Biotage Isolera Four [gradient: 100% hexane for 3 column volumes (CV), then increased to 80:20 hexane:diethyl ether over 15 CV, then increased to 100% diethyl ether over 3 CV] to afford compound **6** as a colourless oil (0.4 g, 60%, *E*:*Z* 1.2:1.0).

Single Step Flow Procedure:
S-(Prop-2-yn-1-yl) ethanethioate **5** (753 mg, 6.6 mmol), thioacetic acid (0.522 mL, 7.3 mmol, 1.1 equiv.), AIBN (0.124 g, 0.753 mmol, 10 mol%) was dissolved in dry CH_2Cl_2 (5 mL) and dry THF (15 mL). The solution was pumped using HPLC K120 Knauer Analytical pump through PTFE tubing (i.d. 0.8 mm, volume: 3.6 mL, residence time: 5.1 minutes) at 0.7 mL/min. The system was fitted with a back-pressure regulator at 40 psi. The first column volume of reactant was discarded, and the following three column volumes were collected and concentrated *in vacuo* at 40 °C. The resulting residue was purified by column chromatography using Biotage Isolera Four [gradient: 100% hexane for 3 column volumes (CV), then increased to 80:20 hexane:diethyl ether over 15 CV, then increased to 100% diethyl ether over 3 CV] to afford compound **6** as a colourless oil (0.35 g, 51%, *E*:*Z* 1.1:1.0).

Combined Flow Procedure for steps (i) and (ii):
Potassium carbonate (8.4 g, 0.06 mol) and sand (11.6 g) were shaken until a homogenous mixture was achieved by eye. The K_2CO_3/sand (20 g) was packed into a glass OmniSep® (150 x 15 mm). The packed column was weighed, and dry tetrahydrofuran (THF) was flowed through at 0.4 mL/min for 1 hour, after which, the column was reweighed. The mass difference equated to the calculated column volume (CV, 8 mL, residence time: 20 minutes).

To an oven dried glass, thioacetic acid (0.4 M) and propargyl bromide (0.4 M) were added to dry THF under argon. The reaction mixture was passed through the base/sand column at 0.4 mL/min using a HPLC pump. The outlet of the base column was connected to one inlet a T-piece mixer. A solution of AIBN (0.04 M), thioacetic acid (0.48 M) in dry THF:CH_2Cl_2 (1:1) under argon was also connected to the T-piece mixer. To the outlet was a PTFE coil (i.d. 0.8 mm) of varying column volumes (CV: 4.74-32 mL), with a back pressure regular (75 psi). The PTFE coil was submerged in a water bath at 85–90°C. The first column volume of reactant was discarded, and the following column volumes were collected and concentrated *in vacuo* at 40 °C. The resulting residue was purified by column chromatography using Biotage Isolera Four [gradient: 100% hexane for 3 column volumes (CV), then increased to 80:20 hexane:diethyl ether over 15 CV, then increased to 100% diethyl ether over 3 CV] to afford compound **6** as a colourless oil (44–61% over 2 steps).

E-isomer: ^{1}H NMR (500 MHz, $CDCl_3$): δ = 6.70 (d, *J* = 9.5 Hz, 1H, $CH_2CH{=}C\mathit{H}$), 5.78-5.86 (m, 1H, $CH_2C\mathit{H}{=}CH$), 3.63 (d, *J* = 7.7 Hz, 2H, $C\mathit{H}_2$), 2.34 (s, 3H, $C\mathit{H}_3$), 2.32 (s, 3H, $C\mathit{H}_3$) ppm.

^{13}C NMR (126 MHz, $CDCl_3$): δ = 194.9 (C=O), 192.7 (C=O), 128.3 ($CH_2CH=$*C*H),120.9 (CH_2*C*H=CH), 31.5 (*C*H_2CH=CH), 30.6 (CH_3), 30.5 (CH_3) ppm.
HRMS (ASAP) $[M+H]^+$ calc. 191.0201, found 191.0203 $[C_7H_{11}O_2S_2]^+$.
IR (neat): 1632, 1352, 1124, 1103, 948, 619 cm^{-1}.

Z-isomer: ^1H NMR (500 MHz, $CDCl_3$): δ = 6.69 (d, *J* = 9.5 Hz, 1H, $CH_2CH=C$*H*), 5.83 (dt, *J* = 9.6, 7.7 Hz, 1H, CH_2C*H*=CH), 3.55 (d, *J* = 7.7 Hz, 2H, C*H_2*), 2.40 (s, 3H, C*H_3*), 2.34 (s, 3H, C*H_3*) ppm.
^{13}C NMR (126 MHz, $CDCl_3$): δ = 195.1 (C=O), 191.1 (C=O), 126.7 ($CH_2CH=$*C*H),120.7 (CH_2*C*H=CH), 31.1 (*C*H_2CH=CH), 30.5 (CH_3), 28.7 (CH_3) ppm.
HRMS (ASAP) $[M+H]^+$ calc. 191.0201, found 191.0203 $[C_7H_{11}O_2S_2]^+$.
IR (neat): 1632, 1352, 1124, 1103, 948, 619 cm^{-1}.

***S*-(3-(Allyldisulfaneyl)allyl) ethanethioate (7)**

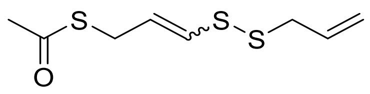

Batch Procedure:
The vinyl thioacetate, *S*,*S*'-(prop-1-ene-1,3-diyl) diethanethioate **6** (100 mg, 0.53 mmol) was dissolved in methanol (5.3 mL) and cooled to –40 °C. Potassium hydroxide (36 mg, 0.56 mmol, 1.05 equiv.) in methanol (5.3 mL) was added to the solution, followed by the direct addition of allyl thiotosylate (121 mg, 0.53 mmol, 1.0 equiv.). The solution was left at –40 °C for 1 hour and warmed to room temperature until thin layer chromatography (TLC) indicated full consumption of starting material. The reaction was quenched with a saturated solution of ammonium chloride (5 mL), and solvent was removed *in vacuo*. The resulting residue was dissolved in diethyl ether (20 mL), and washed with water (20 mL), brine (2 x 20 mL), dried over $MgSO_4$, and concentrated *in vacuo*. The resulting residue was purified using Biotage Isolera Four [gradient: 100% hexane for 3 column volumes (CV), then increased to 80:20 hexane:diethyl ether over 10 CV, held at 20% diethyl ether for 10 CV, then increased to 100% diethyl ether over 3 CV] to afford compound **7** as a pale yellow oil (60 mg, 51%). The bis-disulfide **9** was also isolated as a pale yellow oil (13 mg, 10%).

Flow Procedure:
The vinyl thioacetate, S,S'-(prop-1-ene-1,3-diyl) diethanethioate **6** (100 mg, 0.53 mmol) and allyl thiotosylate (363 mg, 1.59 mmol, 3.0 equiv.) was dissolved in methanol (5.3 mL, 0.1 M) and transferred to a syringe. Potassium hydroxide (69 mg, 1.06 mmol, 2.0 equiv.) was dissolved in methanol (4.5 M) and transferred to a syringe. Both syringes pumped at 0.1 mL/min using syringe pumps through a T-piece mixer and to PTFE tubing (i.d. 0.8 mm, volume: 1 mL, residence time: 5 min). The PTFE coil was submerged in an ice bath at 0 °C. The first column volume of reactant was discarded, and the following seven column volumes were collected and concentrated *in vacuo* at 40 °C. The resulting residue was purified by column chromatography using Biotage Isolera Four [gradient: 100% hexane for 3 column

volumes (CV), then increased to 80:20 hexane:diethyl ether over 10 CV, held at 20% diethyl ether for 10 CV, then increased to 100% diethyl ether over 3 CV] to afford compound **7** as a pale yellow oil (43 mg, 56%). The bis-disulfide **9** was also isolated as a pale yellow oil (20 mg, 10%).

E-isomer: ^{1}H NMR (500 MHz, $CDCl_3$): δ = 6.23 (dt, *J* = 14.7, 1.1 Hz, 1H, $CH_2CH{=}C\mathit{H}$), 5.77-5.88 (m, 1H, $CH_2C\mathit{H}{=}CH$ and $CH_2C\mathit{H}{=}CH_2$), 5.11-5.22 (m, 2H, $CH_2CH{=}C\mathit{H}_2$), 3.58 (dd, *J* = 7.4, 1.1 Hz, 2H, $C\mathit{H}_2CH{=}CH$), 3.30-3.33 (m, 2H, $CH_2CH{=}C\mathit{H}_2$), 2.34 (s, 3H, $C\mathit{H}_3$) ppm.
^{13}C NMR (126 MHz, $CDCl_3$): δ = 194.9 (C=O), 137.2 ($CH_2CH{=}\mathit{C}H_2$), 129.2 ($CH_2CH{=}\mathit{C}H$),125.9 ($CH_2\mathit{C}H{=}CH$), 119.0 ($CH_2CH{=}\mathit{C}H_2$) 41.2 ($\mathit{C}H_2CH{=}CH_2$) 30.8 ($\mathit{C}H_2CH{=}CH$), 30.5 (CH_3) ppm.
HRMS (ASAP) $[M+H]^+$ calc. 221.0128, found 221.0130 $[C_8H_{12}OS_3]^+$.
IR (neat): 1685, 1352, 1130, 1105, 918, 612 cm^{-1}.

Z-isomer: ^{1}H NMR (500 MHz, $CDCl_3$): δ = 6.21 (dt, *J* = 9.2, 1.0 Hz, 1H, $CH_2CH{=}C\mathit{H}$), 5.77-5.88 (m, 1H, $CH_2C\mathit{H}{=}CH$ and $CH_2C\mathit{H}{=}CH_2$), 5.63 (dt, *J* = 9.2, 7.8 Hz, 1H, $CH_2C\mathit{H}{=}CH$), 5.11-5.22 (m, 2H, $CH_2CH{=}C\mathit{H}_2$), 3.65 (dd, *J* = 7.4, 1.1 Hz, 2H, $C\mathit{H}_2CH{=}CH$), 3.34-3.37 (m, 2H, $CH_2CH{=}C\mathit{H}_2$), 2.34 (s, 3H, $C\mathit{H}_3$) ppm.
^{13}C NMR (126 MHz, $CDCl_3$): δ = 195.2 (C=O), 139.3 ($CH_2CH{=}\mathit{C}H$), 132.7 ($CH_2CH{=}\mathit{C}H_2$), 126.6 ($CH_2\mathit{C}H{=}CH$), 119.1 ($CH_2CH{=}\mathit{C}H_2$) 42.0 ($\mathit{C}H_2CH{=}CH_2$) 30.5 (CH_3), 27.2 ($\mathit{C}H_2CH{=}CH$) ppm.
HRMS (ASAP) $[M+H]^+$ calc. 221.0128, found 221.0130 $[C_8H_{12}OS_3]^+$.
IR (neat): 1685, 1352, 1130, 1105, 918, 612 cm^{-1}.

2,2'-(Prop-1-ene-1,3-diyl)bis(1-allyldisulfane) (9)

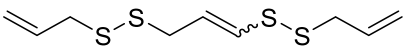

As a mixture of *E*/*Z*-isomers: ^{1}H NMR (400 MHz, $CDCl_3$): δ = 6.32 (dd, *J* = 9.2, 0.6 Hz, 1H), 6.18 (dd, *J* = 14.6, 0.5 Hz, 1H), 5.94 – 5.77 (m, 6H), 5.26 – 5.11 (m, 8H), 3.51 – 3.30 (m, 12H) ppm.
^{13}C NMR (126 MHz, $CDCl_3$): δ = 133.7, 133.5, 133.4, 133.0, 132.8, 129.5, 127.5, 126.3, 119.2, 119.2, 118.9, 118.8, 42.6, 42.4, 42.1, 41.4, 41.0, 36.6 ppm.
HRMS (ASAP) $[M+H]^+$ calc. 251.0057, found 251.0056 $[C_9H_{15}S_4]^+$.
IR (neat): 2361, 2432, 1217, 986, 920, 669, 650 cm^{-1}.
Spectra in accordance with literature.[5]

1-Allyl-2-(3-(allylthio)allyl)disulfane (8)

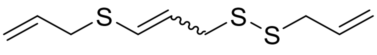

Batch Procedure:

The thioacetate, S-(3-(allyldisulfaneyl)allyl) ethanethioate **7** (100 mg, 0.45 mmol) was dissolved in methanol (4.5 mL) and cooled to –40 °C. Potassium hydroxide (31 mg, 0.48 mmol, 1.05 equiv.) in methanol (4.5 mL) was added to the solution, followed by the direct addition of allyl bromide (54 mg, 0.44 mmol, 1.0 equiv.). The solution was left at –40 °C for 1 hour and warmed to room temperature until thin layer chromatography (TLC) indicated full consumption of starting material. The reaction was quenched with a saturated solution of ammonium chloride (5 mL), and solvent was removed *in vacuo*. The resulting residue was dissolved in diethyl ether (20 mL), and washed with water (20 mL), brine (2 x 20 mL), dried over $MgSO_4$, and concentrated *in vacuo*. The resulting residue was purified using Biotage Isolera Four [gradient: 100% hexane for 10 column volumes (CV), then increased to 90:10 hexane:diethyl ether over 10 CV, then increased to 100% diethyl ether over 3 CV] to afford compound **8** as a pale yellow oil (44 mg, 45%).

Flow Procedure:

The thioacetate, S-(3-(allyldisulfaneyl)allyl) ethanethioate **7** (100 mg, 0.45 mmol) and allyl bromide (163 mg, 1.35 mmol, 3.0 equiv.) was dissolved in methanol (4.5 mL, 0.1 M) and transferred to a syringe. Potassium hydroxide (88 mg, 1.35 mmol, 3.0 equiv.) was dissolved in methanol (4.5 M) and transferred to a syringe. Both syringes pumped at 0.05 mL/min using syringe pumps through a T-piece mixer and to PTFE tubing (i.d. 0.8 mm, volume: 1 mL, residence time: 10 min). The PTFE coil was submerged in an ice bath at 0 °C. The first column volume of reactant was discarded, and the following seven column volumes were collected and concentrated *in vacuo* at 40 °C. The resulting residue was purified by column chromatography using Biotage Isolera Four [gradient: 100% hexane for 10 column volumes (CV), then increased to 90:10 hexane:diethyl ether over 10 CV, then increased to 100% diethyl ether over 3 CV] to afford compound **8** as a pale yellow oil (33 mg, 43%).

Combined Flow Procedure:

Potassium carbonate (9.7 g, 0.07 mol) and sand (14.3 g) were shaken until a homogenous mixture was achieved by eye. The K_2CO_3/sand (24 g, 42 wt.%) was packed into a 10 g Biotage® Isolera column (C1). The packed column was weighed, and dry tetrahydrofuran (THF) was flowed through at 0.4 mL/min for 1 hour, after which, the column was reweighed. The mass difference equated to the calculated column volume (CV, 7.6 mL, residence time: 19 minutes). A solution of thioacetic acid (0.4 M) and propargyl bromide (0.4 M) in THF was passed through the column using a HPLC K120 Knauer Analytical Pump. The outlet was connected a T-piece mixture, where the other inlet was from another HPLC 120 Knauer Analytical pump delivering thioacetic acid (0.4 M) and AIBN (20 mol%) in THF:CH_2Cl_2. The outlet of the two streams was a PTFE coil (C2) with internal diameter 0.8 mm and volume 32 mL with a resulting residence time of 40 minutes from resulting flow rate of 0.8 mL/min. C2 was heated by submerging in a water bath held at 85–90 °C fitted with a back pressure regulator at 75 psi. The outlet of C2 was fed into a cross mixer along with potassium hydroxide solution (0.44 M) and allyl thiotosylate (0.675 M) using a Fusion 100 Touch Syringe Pump. The outlet stream was fed into C3, a PTFE reactor coil of volume 8.7 mL with a residence time of 5.5 minutes. C3 was submerged in an ice bath at 0 °C. In a similar fashion, the outlet of C3 was fed into a cross mixer along with a solution of potassium

hydroxide (0.437 M) and allyl bromide (0.437 M) in methanol using Fusion 100 Touch Syringe Pump. The outlet of which delivered the reaction mixture to C4, a PTFE coil with volume 24 mL and a resulting residence time of 11 minutes. C4 was submerged in an ice bath to allow the reaction to occur at 0 °C. The outlet of the continuous system was collected for 17 minutes (41 mL) and quenched with ammonium chloride. The organic layer was extracted using diethyl ether and washed with water and brine. The organic layer was dried over magnesium sulfate and concentrated *in vacuo.* The resulting residue was purified by column chromatography using Biotage Isolera Four [gradient: 100% hexane for 10 column volumes (CV), then increased to 90:10 hexane:diethyl ether over 10 CV, then increased to 100% diethyl ether over 3 CV] to afford compound **8** as a pale yellow oil (73 mg, 12%).

E-isomer: ^{1}H NMR (500 MHz, $CDCl_3$): δ = 6.12 (dt, *J* = 15.1, 1.0 Hz, 1H, $CH_2CH{=}C\textit{H}$), 5.77-5.94 (m, 2H, $CH_2C\textit{H}{=}CH_2$ and $CH_2C\textit{H}{=}CH_2$), 5.60 (dt, *J* = 15.1, 7.7 Hz, 1 H, $CH_2C\textit{H}{=}CH$), 5.11-5.22 (m, 4H, $CH_2CH{=}C\textit{H}_2$ and $CH_2CH{=}C\textit{H}_2$), 3.37 (dd, *J* = 7.7, 1.0 Hz, 2H, $C\textit{H}_2CH{=}CH$), 3.33 (dt, *J* = 7.2, 1.2 Hz, 2H, $C\textit{H}_2CH{=}CH_2$), 3.30-3.32 (m, 2H, $C\textit{H}_2CH{=}C\textit{H}_2$) ppm.
^{13}C NMR (126 MHz, $CDCl_3$): δ = 133.6 ($CH_2\textit{C}H{=}CH_2$), 133.5 ($CH_2\textit{C}H{=}CH_2$), 127.7 ($CH_2CH{=}\textit{C}H$),123.5 ($CH_2\textit{C}H{=}CH$), 118.6 ($CH_2CH{=}\textit{C}H_2$), 117.9 ($CH_2CH{=}\textit{C}H_2$), 42.7 ($\textit{C}H_2CH{=}CH_2$), 42.1 ($\textit{C}H_2CH{=}CH$), 35.7 ($\textit{C}H_2CH{=}CH_2$) ppm.
HRMS (ASAP) $[M+H]^+$ calc. 219.0336 found 219.0336 $[C_9H_{15}OS_3]^+$.
IR (neat): 3080, 2978, 1633, 1423, 1205, 985, 916, 756 cm^{-1}.

Z-isomer: ^{1}H NMR (500 MHz, $CDCl_3$): δ = 6.15 (dt, *J* = 9.5, 0.9 Hz, 1H, $CH_2CH{=}C\textit{H}$), 5.77-5.94 (m, 2H, $CH_2C\textit{H}{=}CH_2$ and $CH_2C\textit{H}{=}CH_2$), 5.70 (dt, *J* = 9.5, 0.9 Hz, 1 H, $CH_2C\textit{H}{=}CH$), 5.11-5.22 (m, 4H, $CH_2CH{=}C\textit{H}_2$ and $CH_2CH{=}C\textit{H}_2$), 3.37 (dd, *J* = 7.7, 1.0 Hz, 2H, $C\textit{H}_2CH{=}CH$), 3.33 (dt, *J* = 7.2, 1.2 Hz, 2H, $C\textit{H}_2CH{=}CH_2$), 3.30-3.32 (m, 2H, $C\textit{H}_2CH{=}C\textit{H}_2$) ppm.
^{13}C NMR (126 MHz, $CDCl_3$): δ = 134.1 ($CH_2\textit{C}H{=}CH_2$), 133.5 ($CH_2\textit{C}H{=}CH_2$), 128.9 ($CH_2CH{=}\textit{C}H$),124.8 ($CH_2\textit{C}H{=}CH$), 118.7 ($CH_2CH{=}\textit{C}H_2$), 117.8 ($CH_2CH{=}\textit{C}H_2$), 42.3 ($\textit{C}H_2CH{=}CH_2$), 37.5 ($\textit{C}H_2CH{=}CH$), 36.8 ($\textit{C}H_2CH{=}CH_2$) ppm.
HRMS (ASAP) $[M+H]^+$ calc. 219.0336 found 219.0336 $[C_9H_{15}OS_3]^+$.
IR (neat): 3080, 2978, 1633, 1423, 1205, 985, 916, 756 cm^{-1}.

_S_-(3-((4-Methoxybenzyl)thio)allyl) ethanethioate (14)

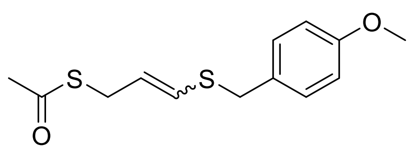

S-(Prop-2-yn-1-yl) ethanethioate **5** (3.64 g, 32 mmol) was dissolved in dry toluene (20 mL) and the solution was heated to 85 °C under argon atmosphere. Azobisisobutyronitrile (AIBN) (1.05 g, 6.4 mmol, 0.2 equiv.) was added to the solution directly, followed by the dropwise addition of *p*-methoxy benzyl mercaptan (7.4 mL) in toluene (20 mL) over 45 minutes using a syringe pump. The mixture was left to stir at 85 °C for a further 1 hour. The reaction was then quenched with aqueous saturated solution of sodium carbonate (20 mL) and the toluene was removed *in vacuo*. The remaining residue was dissolved

diethyl ether (20 mL) and the organic layer was washed with brine (2 x 10 mL) and dried over $MgSO_4$. The solvent was evaporated *in vacuo* and the resulting residue was purified by column chromatography using Biotage Isolera Four [gradient: 100% hexane for 3 column volumes (CV), then increased to 80:20 hexane:diethyl ether over 15 CV, then increased to 100% diethyl ether over 3 CV] to afford compound **14** as a pale yellow oil (6.13 g, 71%).

As a mixture of *E*/Z isomers: ^{1}H NMR (500 MHz, $CDCl_3$): δ = 7.25 – 7.21 (m, 4H, Ar*H*), 6.87 – 6.83 (m, 4H, Ar*H*), 6.20 (dt, J = 15.0, 1.1 Hz, 1H, HC=C*H*S), 6.08 (dt, J = 9.3, 0.9 Hz, 1H, HC=C*H*S), 5.64 – 5.50 (m, 2H, SCH_2C*H*=CH), 3.85 (s, 2H, SCH_2PMP), 3.83 (s, 2H, SCH_2PMP), 3.80 (s, J = 2.1 Hz, 6H, OCH_3), 3.60 (dd, J = 7.7, 0.9 Hz, 2H, SCH_2CH=CH), 3.53 (dd, J = 7.4, 1.1 Hz, 2H, SCH_2CH=CH), 2.32 (s, 3H, CH_3COS), 2.32 (s, 3H, CH_3COS) ppm.
^{13}C NMR (126 MHz, $CDCl_3$): δ = 195.7 (*C*=O), 195.3 (*C*=O), 159.0 ($C_{Ar}OCH_3$), 158.9 ($C_{Ar}OCH_3$), 130.1 (C_{Ar}), 130.0 (C_{Ar}), 129.7 (C_{Ar}), 129.2 (C_{Ar}), 128.5 (CH=CHS), 127.9 (CH=*C*HS), 123.9 (*C*H=CHS), 123.1 (*C*H=CHS), 114.2 (C_{Ar}), 114.1 (C_{Ar}), 55.4 (O*C*H_3), 37.6 (S*C*H_2C_{Ar}), 36.6 (S*C*H_2C_{Ar}), 31.8 (S*C*H_2CH=CH), 30.6 (S*C*H_2CH=CH), 30.5 (*C*H_3C=O), 27.9 (*C*H_3C=O) ppm.
HRMS (EI) $[M+H]^+$ calc. 268.05862, found 286.0587 $[C_{13}H_{16}O_2S_2]^+$.
IR (neat): 2835, 1686, 1511, 1248, 1132, 1033, 956, 834, 626 cm^{-1}.

Allyl(3-((4-methoxybenzyl)thio)allyl)sulfane (15)

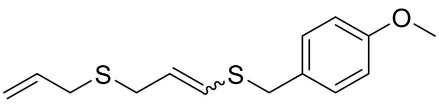

The thioacetate **14** (4.68 g, 17.5 mmol) was dissolved in methanol (100 mL) and cooled to -40 °C. Potassium hydroxide (1.7 g, 26.3 mmol, 1.5 equiv.) was added to the solution, followed by the direct addition of allyl bromide (3 mL, 35 mmol, 2 equiv.). The solution was left at -40 °C for 1 hour and warmed to room temperature until thin layer chromatography (TLC) indicated full consumption of starting material. The reaction was quenched with a saturated solution of ammonium chloride (10 mL), and solvent was removed *in vacuo*. The resulting residue was dissolved in diethyl ether (20 mL), and washed with water (20 mL), brine (2 x 20 mL), dried over $MgSO_4$, and concentrated *in vacuo*. The resulting residue was purified using Biotage Isolera Four [gradient: 100% hexane for 10 column volumes (CV), then increased to 90:10 hexane:diethyl ether over 10 CV, then increased to 100% diethyl ether over 3 CV] to afford compound **15** as a pale yellow oil (3.5 g, 88%).

As a mixture of *E/Z* isomers: ^{1}H NMR (500 MHz, $CDCl_3$): δ = 7.26 – 7.19 (m, 4H, Ar*H*), 6.85 (dd, J = 8.7, 2.7 Hz, 4H, Ar*H*), 6.07 (d, J = 9.4 Hz, 1H, HC=C*H*S), 6.01 (d, J = 15.0 Hz, 1H, HC=C*H*S), 5.85 – 5.67 (m, 2H, CH_2=C*H*CH_2), 5.62 – 5.51 (m, 2H, CH_2=C*H*CH_2), 5.18 – 4.96 (m, 4H, CH_2=CHCH_2S), 3.85 (s, 2H, SCH_2PMP), 3.83 (s, 2H, SCH_2PMP), 3.79 (s, 6H, OCH_3), 3.18 (d, J = 7.5 Hz, 2H, SCH_2CH=CH), 3.09 (d, J = 7.3 Hz, 2H, SCH_2CH=CH), 3.05 (d, J = 7.1 Hz, 2H, CH_2=CHCH_2S), 3.00 (d, J = 7.1 Hz, 2H, CH_2=CHCH_2S) ppm.

^{13}C NMR (126 MHz, $CDCl_3$): δ = 159.0 ($C_{Ar}OCH_3$), 134.4 (CH_2=CHCH_2S), 130.1 (C_{Ar}), 130.0 (C_{Ar}), 129.9 (C_{Ar}), 129.4 (C_{Ar}), 127.2 (C_{Ar}), 126.3 (C_{Ar}), 126.0 (CH=CHS), 125.2 (CH=CHS), 117.3 ($C$$H_2$=CH$CH_2$S), 117.2, ($C$$H_2$=CH$CH_2$S), 114.2 ($C_{Ar}$), 55.4 (O$CH_3$), 55.4 (O$CH_3$), 37.7 (S$CH_2C_{Ar}$), 36.7 (S$CH_2C_{Ar}$), 34.3 (CH$CH_2$S), 33.5 (CH$CH_2$S), 33.0 (S$CH_2$CH), 29.4 (S$CH_2$CH) ppm.
HRMS (ES) $[M+H]^+$ calc. 267.0877, found 267.0879 $[C_{14}H_{19}O_S2]^+$.
IR (neat): 2835, 1610, 1511, 1249, 1176, 1034, 918, 832 cm^{-1}.

***S*-(3-(Allylthio)prop-1-en-1-yl) ethanethioate (16)**

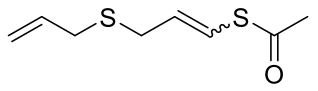

Sulfide **15** (2.26 g, 8.5 mmol) was dissolved in anhydrous CH_2Cl_2 (40 mL) under argon atmosphere and cooled to –78 °C. A premixed solution of acetic acid (3.75 mL, 65.6 mmol, 7.7 equiv.) and trifluoacetic anhydride (9.2 mL, 65.6 mmol, 7.7 equiv.) was added dropwise and the reaction left to stir at –78°C for a further 2 hours. The reaction was quenched with a saturated solution of sodium carbonate (20 mL) and the product was extracted using CH_2Cl_2 (3 x 20 mL). The combined organic layers were washed with brine (20 mL), water (20 mL) and dried over magnesium sulfate and concentrated *in vacuo*. The resulting residue was purified using Biotage Isolera Four [gradient: 100% hexane for 5 column volumes (CV), then increased to 80:20 hexane:diethyl ether over 10 CV, then increased to 100% diethyl ether over 3 CV] to afford compound **16** as a pale yellow oil (329 mg, 21%).

^{1}H NMR (500 MHz, $CDCl_3$): δ = 6.68 (dt, J = 9.6, 1.1 Hz, 1H, CH_2=CHS), 6.53 (dt, J = 15.6, 1.2 Hz, 1H, CH_2=CHS), 5.88 – 5.69 (m, 4H, CH_2=C$H$$CH_2$ and SCH_2CH=CH), 5.22 – 5.05 (m, 4H, CH_2=CH), 3.19 (dd, J = 7.4, 1.2 Hz, 2H, SCH_2CH=CH), 3.14 (dd, J = 7.6, 1.1 Hz, 2H, SCH_2CH=CH), 3.12 – 3.09 (m, 2H, CHCH_2S), 3.06 – 3.01 (m, 2H, CHCH_2S), 2.36 (s, J = 3.9 Hz, 3H, CH_3), 2.35 (s, 3H, CH_3) ppm.
HRMS (CI) $[M+H]^+$ calc. 188.03241, found 188.0323 $[C_8H_{12}OS_2]^+$.
IR (neat): 2835, 1610, 1511, 1249, 1176, 1034, 918, 832 cm^{-1}.

1-Allyl-2-(3-(allylthio)prop-1-en-1-yl)disulfane (12)

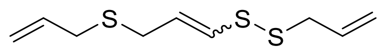

Thioacetate **16** (52 mg, 0.28 mmol) was dissolved in methanol (2.8 mL) and cooled to –40 °C. Potassium hydroxide (20 mg, 0.31 mmol, 1.1 equiv.) in methanol (2 mL) was added to the solution, followed by the addition of allyl thiotosylate (76.7 mg, 0.34 mmol, 1.2 equiv.). The solution was left at – 40 °C for 1 hour and warmed to room temperature until thin layer chromatography (TLC) indicated full consumption of starting material. The reaction was quenched with a saturated solution of ammonium chloride (5 mL), and solvent was removed *in vacuo*. The resulting residue was dissolved in diethyl ether

(20 mL), and washed with water (20 mL), brine (2 x 20 mL), dried over $MgSO_4$, and concentrated *in vacuo*. The resulting residue was purified using Biotage Isolera Four [gradient: 100% hexane for 10 column volumes (CV), then increased to 90:10 hexane:diethyl ether over 10 CV, then increased to 100% diethyl ether over 3 CV] to afford compound **12** as a pale yellow oil (43 mg, 70%).

1-Allyl-2-(3-(allylsulfinyl)prop-1-en-1-yl)disulfane (3)

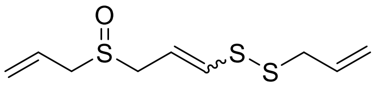

Sulfide **12** (43 mg, 0.2 mmol) was dissolved in dry CH_2Cl_2 (2 mL) and cooled to –78 °C. Recrystallised *m*CPBA (35 mg, 0.2 mmol, 1 equiv.) was added to the solution and left at –78 °C for one hour, then warmed to room temperature until thin layer chromatography (TLC) indicated full consumption of starting material. The reaction was quenched with a saturated solution of sodium carbonate (5 mL), and the product was extracted using CH_2Cl_2 (3 x 10 mL). The combined organic layers were washed with water (20 mL), brine (20 mL), dried over magnesium sulfate and concentrated *in vacuo*. The resulting residue was purified using Biotage Isolera Four [gradient: 100% hexane for 2 column volumes (CV), then increased to 20:80 hexane:diethyl ether over 3 CV, then increased to 100% diethyl ether over 10 CV, and held at 100% diethyl ether for 20 CV] to afford compound **3** as a pale yellow oil (23 mg, 49%). Spectra in accordance with literature.[6]

^{1}H NMR (500 MHz, $CDCl_3$): δ = 6.55 (dt, J = 9.5, 1.0 Hz, 1H, *E*-CH=C*H*SS), 6.36 (dt, J = 14.8, 1.1 Hz, 1H, *Z*-CH=C*H*SS), 5.95 – 5.70 (m, 6H, CH=C*H*C*H*$_2$), 5.47 – 5.36 (m, 4H, CH_2=CHC*H*$_2$S=O), 5.20 – 5.13 (m, 4H, C*H*$_2$=CHCH$_2$S), 3.59 – 3.42 (m, 8H, C*H*$_2$S(=O)C*H*$_2$), 3.39 (d, J = 7.5 Hz, 2H, SSC*H*$_2$), 3.34 (d, J = 7.3 Hz, 2H, SSC*H*$_2$) ppm.
^{13}C NMR (126 MHz, $CDCl_3$): δ = 138.7 (S(=O)CH$_2$*C*H=CH), 134.8 (S(=O)CH$_2$*C*H=CH), 132.7 (SSCH$_2$*C*H=CH$_2$), 132.6 (SSCH$_2$*C*H=CH$_2$), 125.8 (CH$_2$=*C*HCH$_2$S), 125.7 (CH$_2$=*C*HCH$_2$S), 124.0 (*C*H$_2$=CHCH$_2$S), 123.9 (*C*H$_2$=CHCH$_2$S), 119.4 (SSCH$_2$CH*C*H$_2$), 119.4 (SSCH$_2$CH*C*H$_2$), 118.2 (CH=CHSS), 116.9 (CH=*C*HSS), 55.0 (*C*H$_2$S=O), 54.5 (*C*H$_2$S=O), 53.1 (S(=O)*C*H$_2$), 49.7 (S(=O)*C*H$_2$), 42.2 (SS*C*H$_2$), 41.4 (SS*C*H$_2$) ppm.

Design of Experiment for Step (iii) and (iv)

Umetrics MODDE Pro 12.1 was used for the design of experiment software. The ‘Design Wizard’ was used to create an optimised design that investigated two quantitative factors with two levels (time and temperature) and two quantitative factors with three levels (equivalence of electrophile and base). Umetric MODDE Pro 12.1 produced a randomised design of 21 experiments, including 3 repeats. The equivalence of electrophile and base were controlled by the concentration whilst keeping the flow rate constant. The first one and a half column volumes of each run was discarded and then approximately 750 μL of solution was collected into a 1 mL HPLC vial containing 3 drops of saturated solution of ammonium chloride. From the quenched reaction mixture, a 50 μL aliquot was diluted into 1 mL acetonitrile and analysed by Reverse Phase HPLC. The solvent system for the starting material was chosen as a mixture of THF, dichloromethane and methanol to best represent the final system. Modde software based on the results analysed by HPLC predicted that at 2.0 equiv. of base and 3.0 equiv. of allyl thiotosylate at 0 °C and 5 minutes residence time, **7** could be obtained as in 54%, with 41% of **9** and unreacted 3% of **6** by integrated peak area at 254 nm. Modde software based on the results analysed by HPLC predicted that at 3.0 equiv. of base and 3.0 equiv. of allyl bromide at 0 °C and 10 minute residence time, **7** could be obtained in <60% as a percentage of peak area at 254 nm.

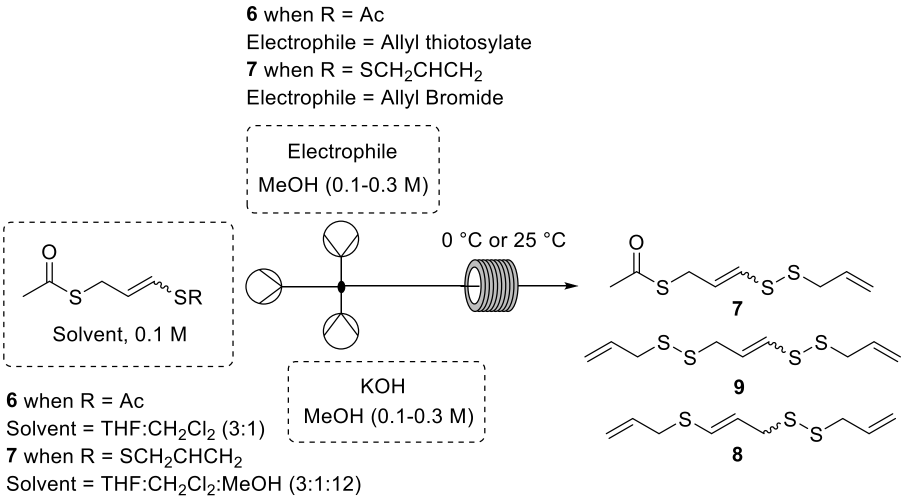

Scheme 1: Design of experiments Set-Up. Flow rate: 3 x 0.03333 mL/min to achieve 10 minutes residence time or 3 x 0.06666 mL/min to achieve 5 minutes residence time. Column: PTFE i.d. 0.8 mm, CV: 1 mL submerged in ice bath at 0 °C or water bath at 25 °C. HPLC isocratic Method: C18 4.6 x 150 mm, 5 micron; solvent A (30%): water 0.1% trifluoroacetic acid; solvent B (70%): acetonitrile 0.1% trifluoroacetic acid; flow rate: 1.5 mL/min; stop time: 15.0 min; injection volume: 20.0 μL; temperature: 30.0 °C; wavelength: 254 nm.

Run Order	Experiment Number	Res. Time (min.)	Temp. (°C)	Electrophile (equiv.)	KOH (equiv.)
1	5	10	25	2	1
2	15	5	0	3	3
3	17	10	0	2	3
4	16	10	25	1	3
5	1	5	25	1	1
6	9	10	25	3	2
7	21	10	0	1	2
8	8	10	0	2	2
9	11	5	25	2	2
10	12	5	0	3	2
11	18	10	25	3	3
12	14	5	25	2	3
13	10	5	25	1	2
14	13	5	0	1	3
15	2	5	0	2	1
16	4	10	0	1	1
17	3	5	25	3	1
18	6	10	0	3	1
19	7	10	0	1	2
20	20	5	25	2	2
21	19	10	25	3	3

Table 1: Design of Experiments Table for step (iii) where electrophile is allyl thiotosylate and step (iv) where electrophile is allyl bromide.

NMR Spectra

***S*,*S*'-(Prop-1-ene-1,3-diyl) diethanethioate (6)**

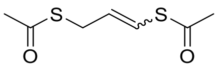

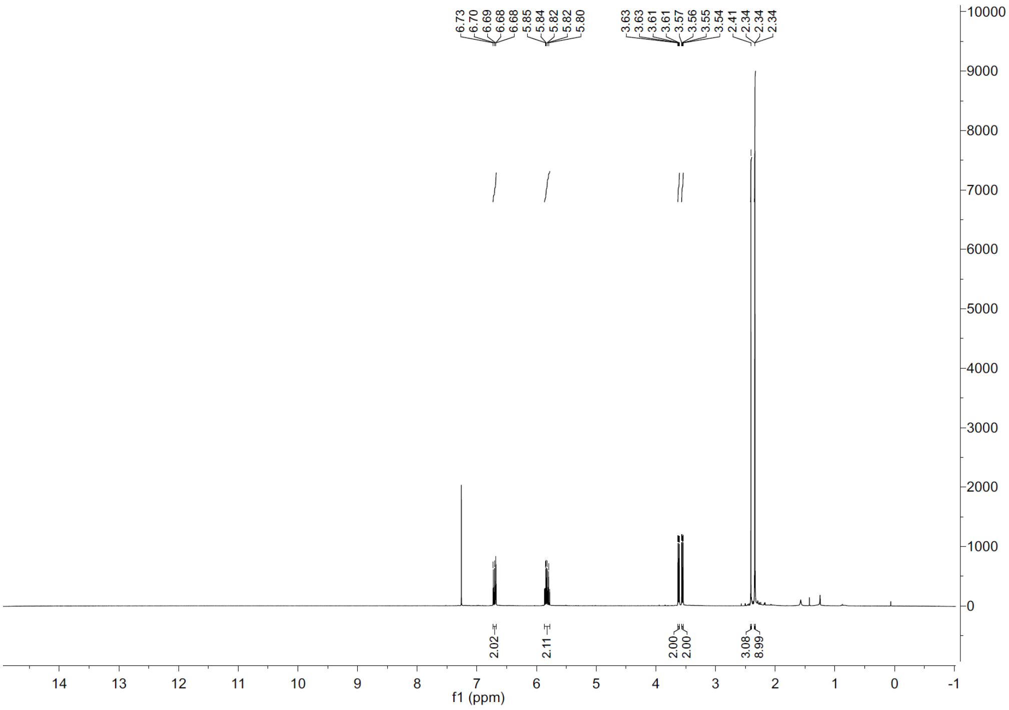

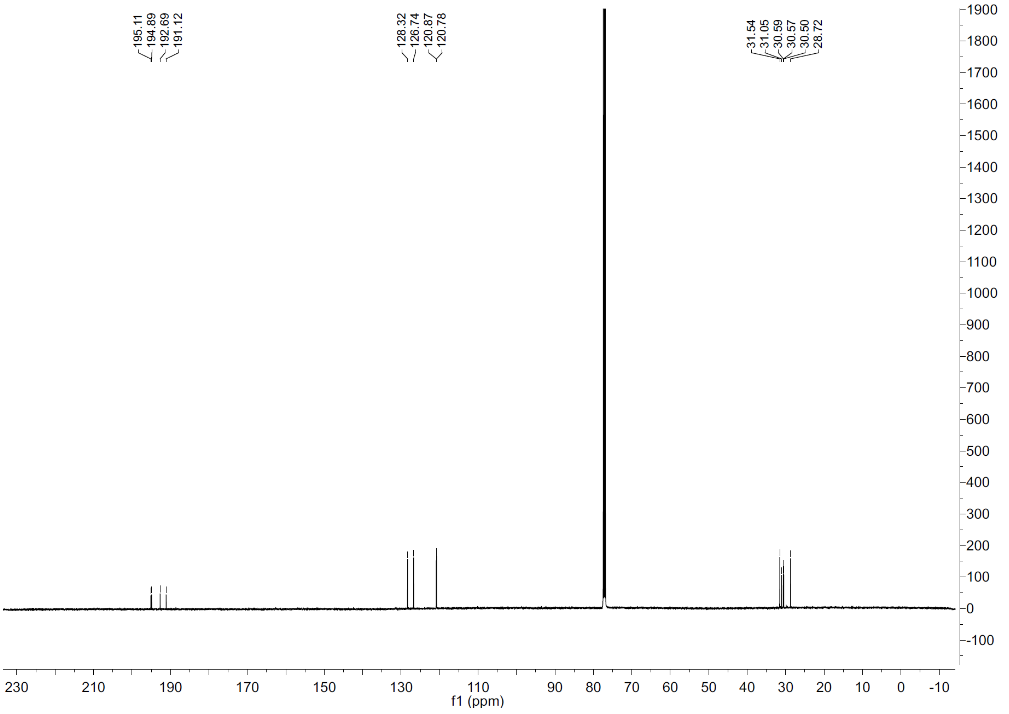

S-(3-(Allyldisulfaneyl)allyl) ethanethioate (7)

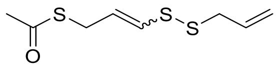

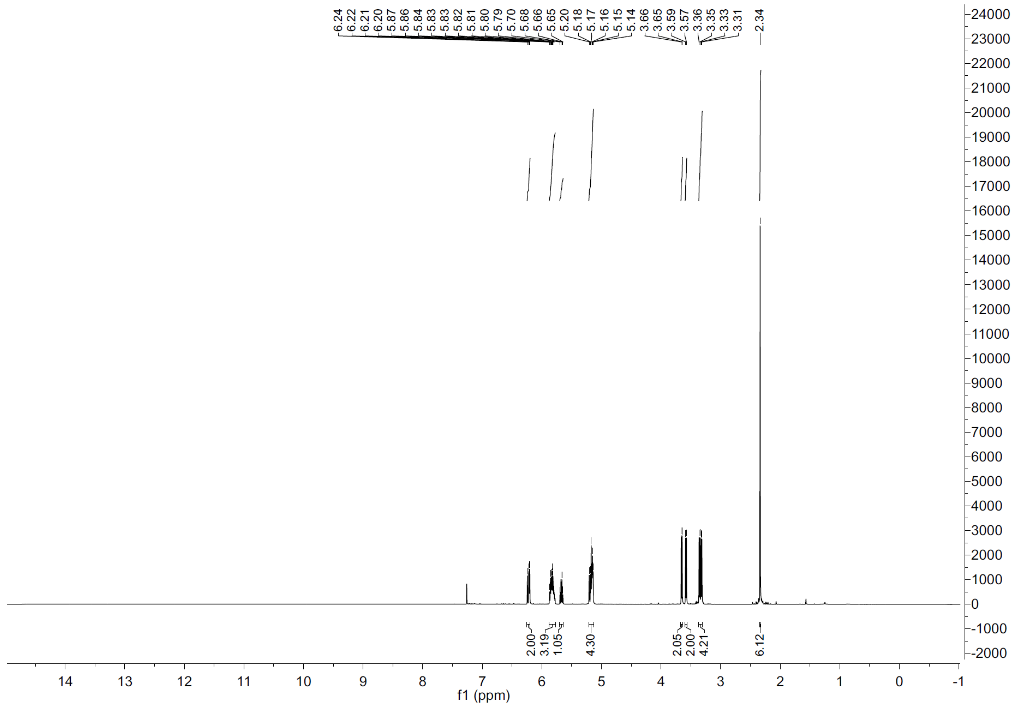

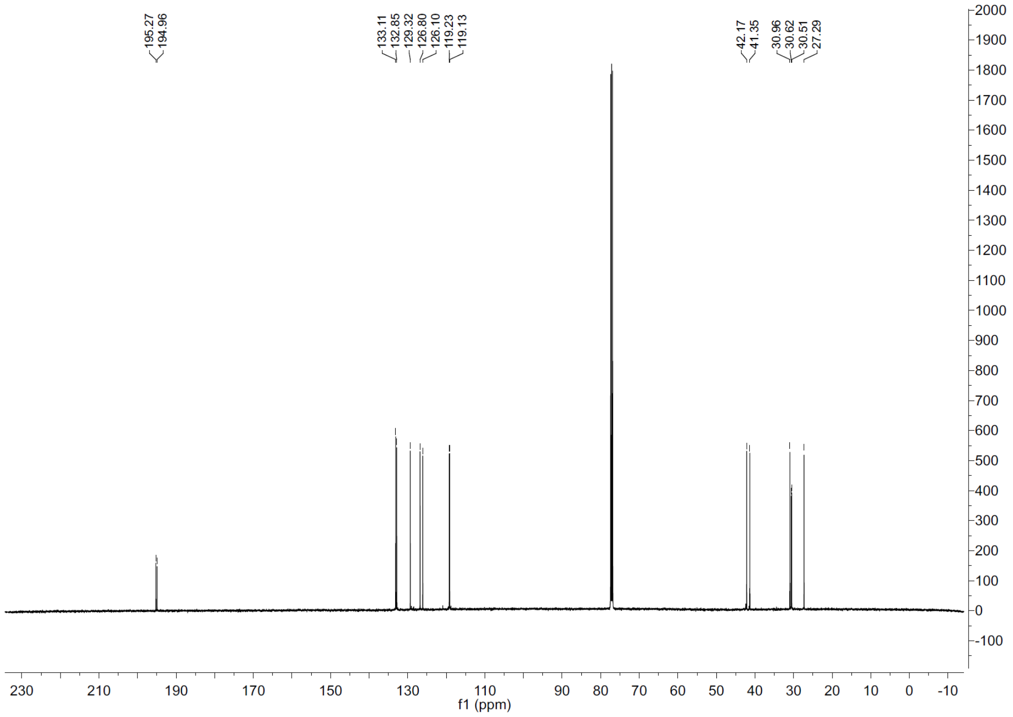

1-Allyl-2-(3-(allylthio)prop-1-en-1-yl)disulfane (8)

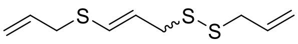

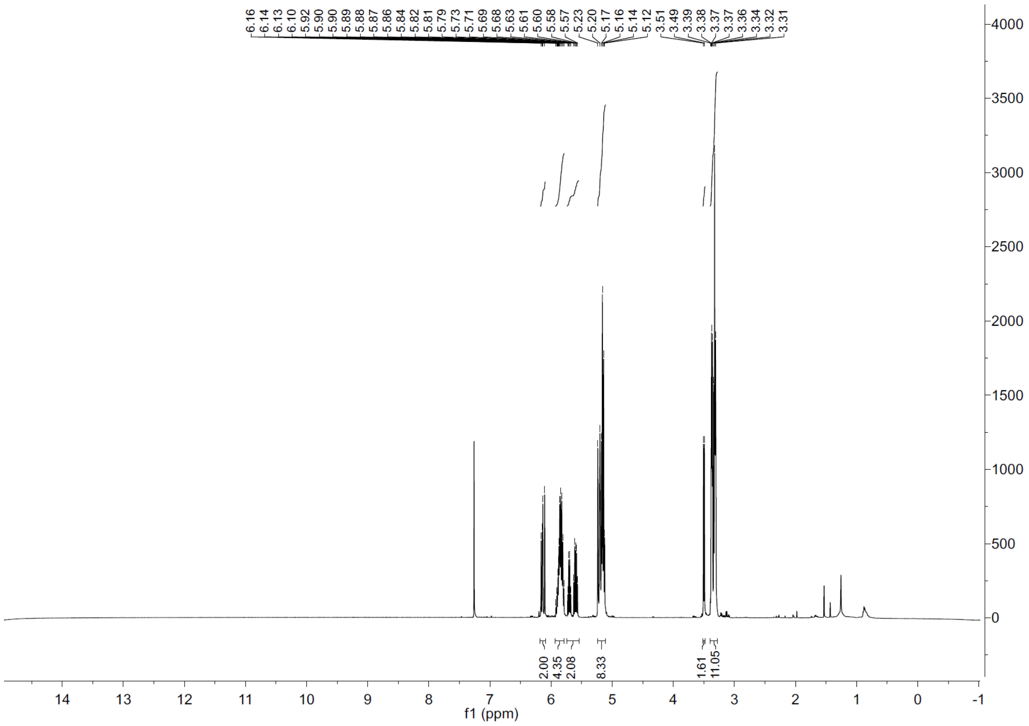

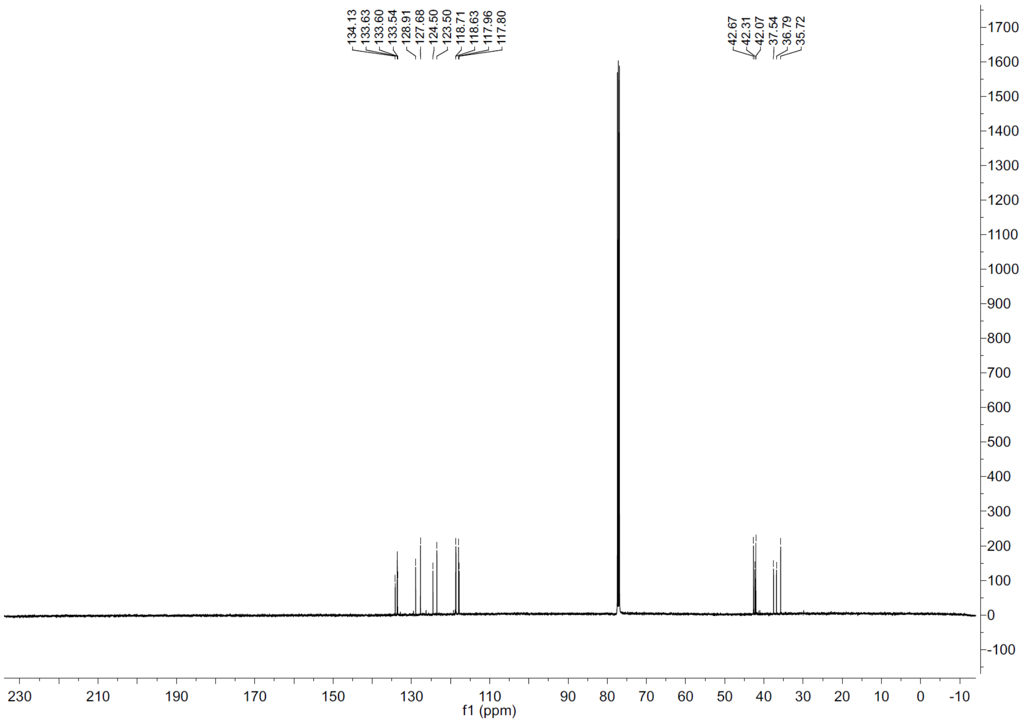

S-(3-((4-Methoxybenzyl)thio)allyl) ethanethioate (14)

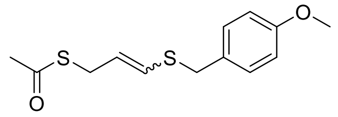

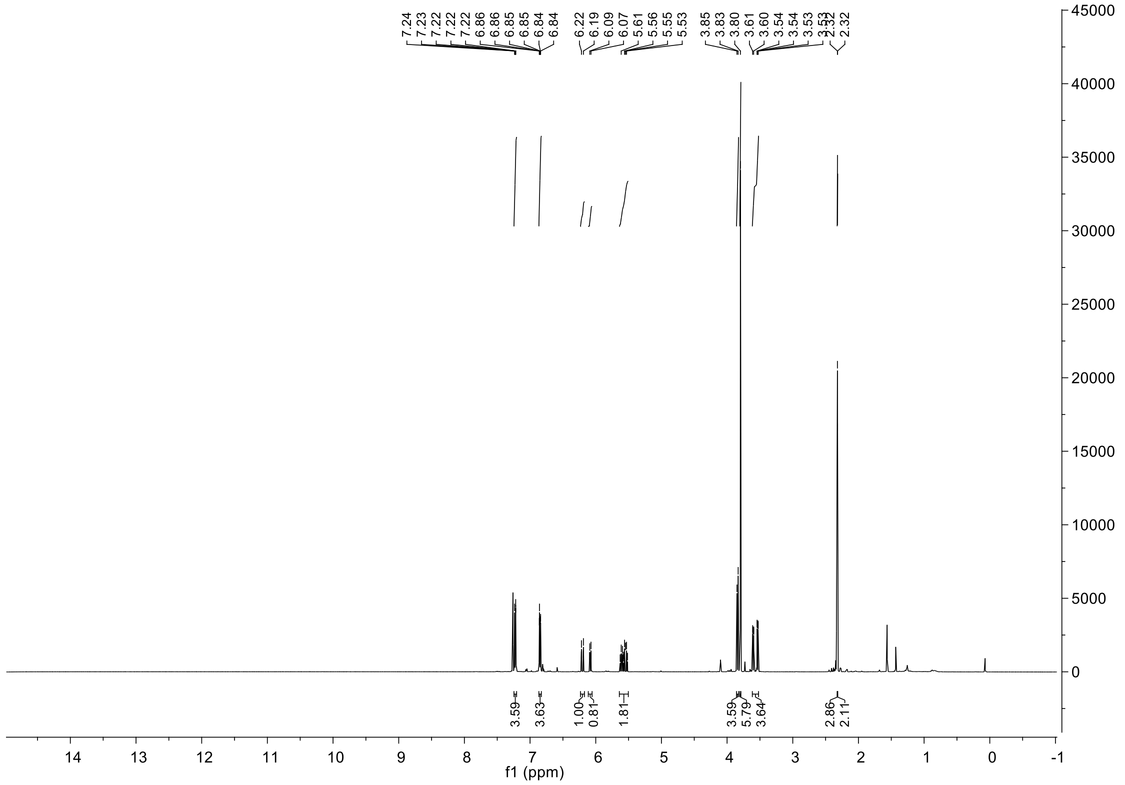

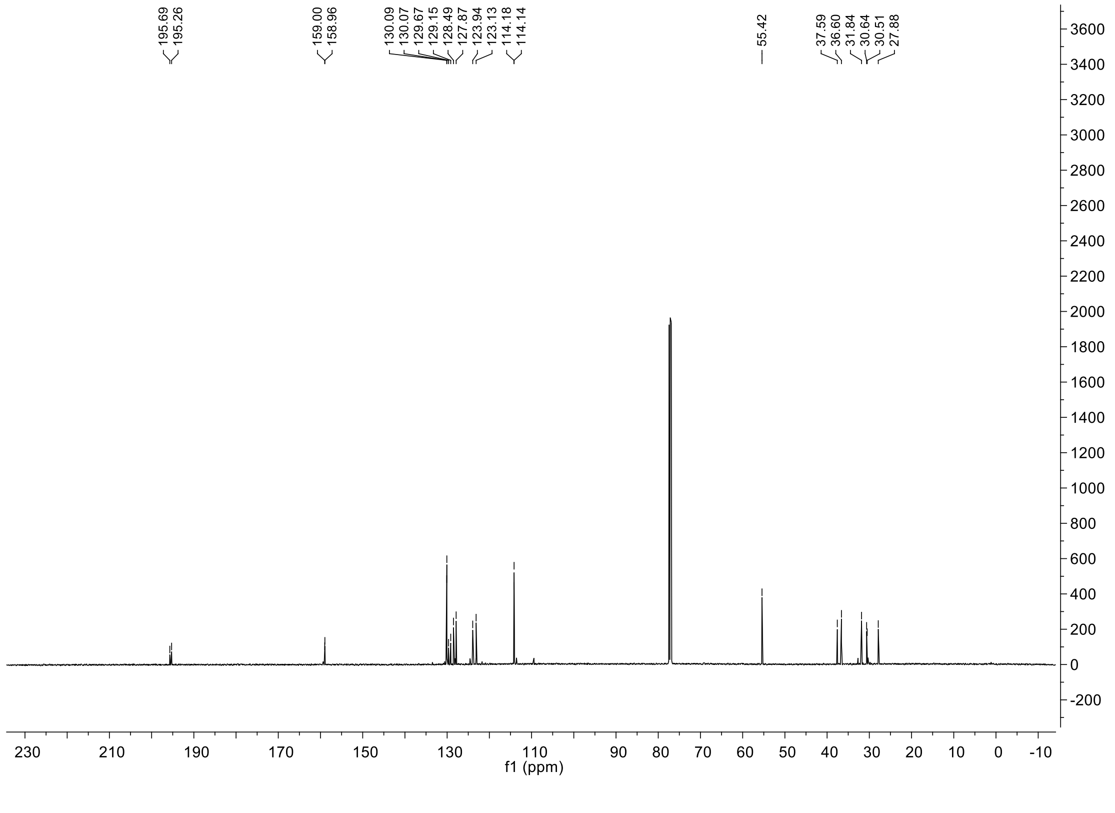

Allyl(3-((4-methoxybenzyl)thio)allyl)sulfane (15)

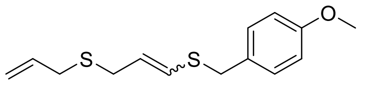

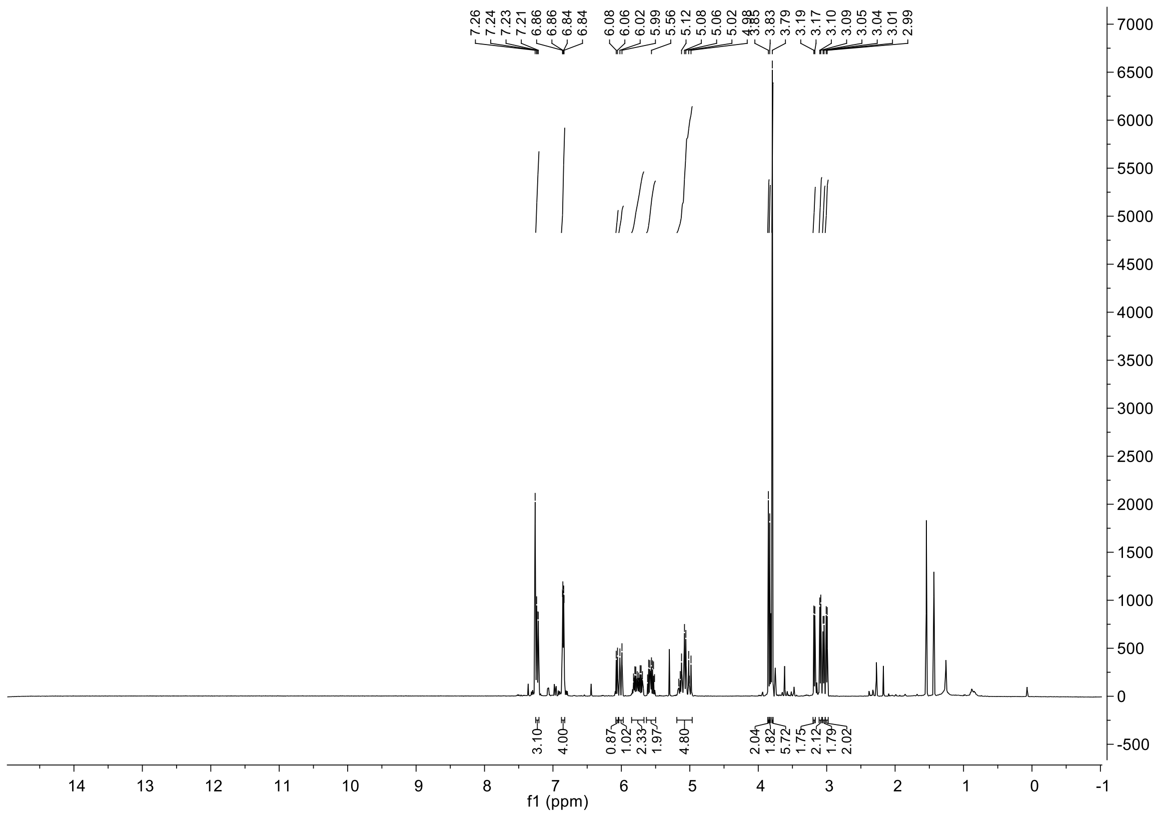

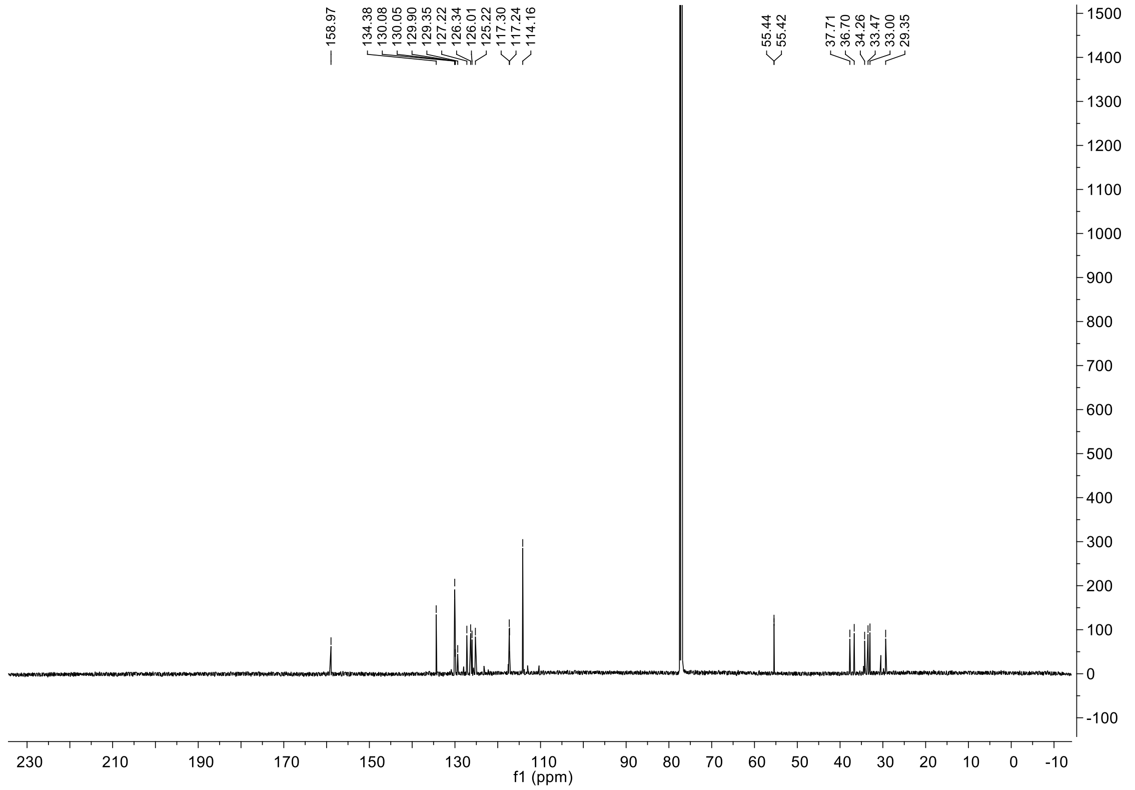

3-(Allyldisulfaneyl)-2-(allylthio)propanal (13)

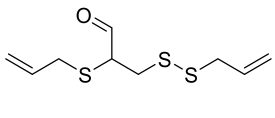

^{1}H NMR (500 MHz, $CDCl_3$): δ = 8.04 (d, J = 9.7 Hz, 1H, C*H*O), 5.85 (td, J = 17.0, 7.5 Hz, 1H, CH_2=C*H*CH_2S), 5.75 (dt, J = 16.9, 7.1 Hz, 1H, CH_2=C*H*CH_2S), 5.25 – 5.09 (m, 4H, CH_2=CHCH_2S and SCH_2CH=C*H*$_2$), 4.08 – 3.99 (m, 1H, SC*H*CHO), 3.36 (d, J = 7.3 Hz, 2H, SCHC*H*$_2$SS), 2.87 (ddd, J = 20.9, 13.4, 6.8 Hz, 2H, SSC*H*$_2$), 2.51 – 2.25 (m, 2H, CHC*H*$_2$S) ppm.

^{13}C NMR (126 MHz, $CDCl_3$): δ = 178.5 (*C*HO), 133.9 (CH_2=*C*HCH_2), 133.5 (CH_2=*C*HCH_2), 118.9 (*C*H_2=CH), 118.6 (*C*H_2=CH), 42.4 (SS*C*H_2CH), 42.1 (S*C*HCH_2), 37.3 (SCH*C*H_2), 36.6 (CH*C*H_2S) ppm.

HRMS (EI) $[M+H]^+$ calc. 234.0207, found 234.0204 $[C_9H_{14}OS_3]^+$.

IR (neat): 2916, 2360, 1636, 1422, 1217, 1125, 988, 916, 750, 579 cm^{-1}.

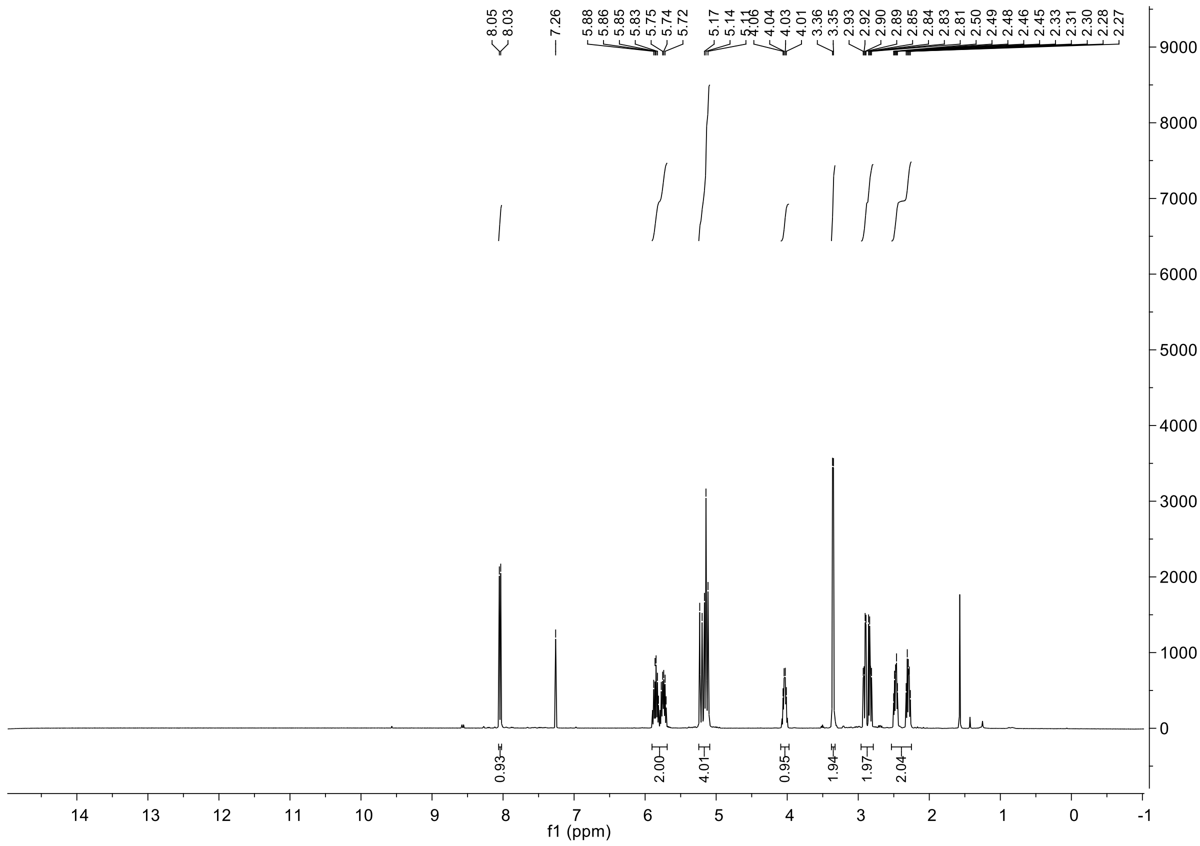

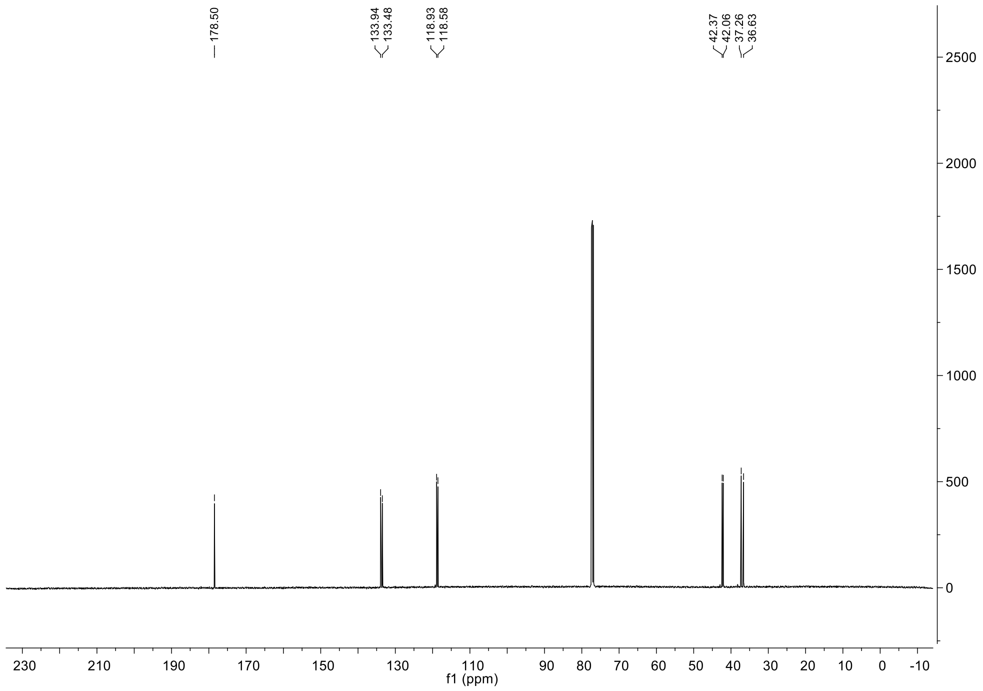

Control experiment to determine rearrangement of vinylic sulfide

In order to validate the configuration of **13**, the vinylic sulfide **17** was synthesised from compound **6** using potassium hydroxide and allyl bromide. Compound **17** was oxidised to **18**.

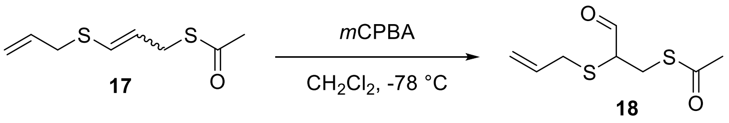

***S*-(3-(Allylthio)allyl) ethanethioate (17)**

^{1}H NMR (500 MHz, $CDCl_3$): δ = 6.17 (dt, *J* = 15.0, 1.1 Hz, 1H, SC*H*=CH), 6.07 (dt, *J* = 9.4, 0.9 Hz, 1H, SC*H*=CH), 5.82 (ddtd, *J* = 11.7, 8.6, 7.0, 1.6 Hz, 2H, CH_2=C*H*CH_2S), 5.65 (dt, *J* = 9.4, 7.7 Hz, 1H, SCH=C*H*), 5.57 (dt, *J* = 14.9, 7.4 Hz, 1H, SCH=C*H*), 5.21 – 5.16 (m, 2H, C*H*$_2$=CHCH_2S), 5.16 – 5.11 (m, 2H, C*H*$_2$=CHCH_2S), 3.63 (d, *J* = 7.7, 0.9 Hz, 2H, CH=CHC*H*$_2$S), 3.35 (d, *J* = 7.4, 1.1 Hz, 2H, CH=CHC*H*$_2$S), 3.32 – 3.27 (m, 4H, CH_2=CHC*H*$_2$S), 2.33 (s, 3H, C*H*$_3$), 2.32 (s, 3H, C*H*$_3$) ppm.
^{13}C NMR (126 MHz, $CDCl_3$): δ = 195.7 (S*C*=OCH_3), 195.2 (S*C*=OCH_3), 134.0 (CH_2=*C*HCH_2S), 133.5 (CH_2=*C*HCH_2S), 128.3 (CH*2*=CHCH_2S), 127.4 (CH*2*=*C*HCH_2S), 123.7 (*C*H$_2$=CH), 123.3 (*C*H$_2$=CH), 117.9 (SCH=*C*H), 117.9 (SCH=*C*H), 36.5 (CH*C*H$_2$S), 35.6 (CH*C*H$_2$S), 31.8 (*C*H$_2$SCO), 30.6 (*C*H$_2$SCO), 30.5 (*C*H$_3$), 28.0 (*C*H$_3$) ppm.
HRMS (CI) $[M+H]^+$ calc. 188.03241, found 188.0323 $[C_8H_{12}OS_2]^+$.
IR (neat):

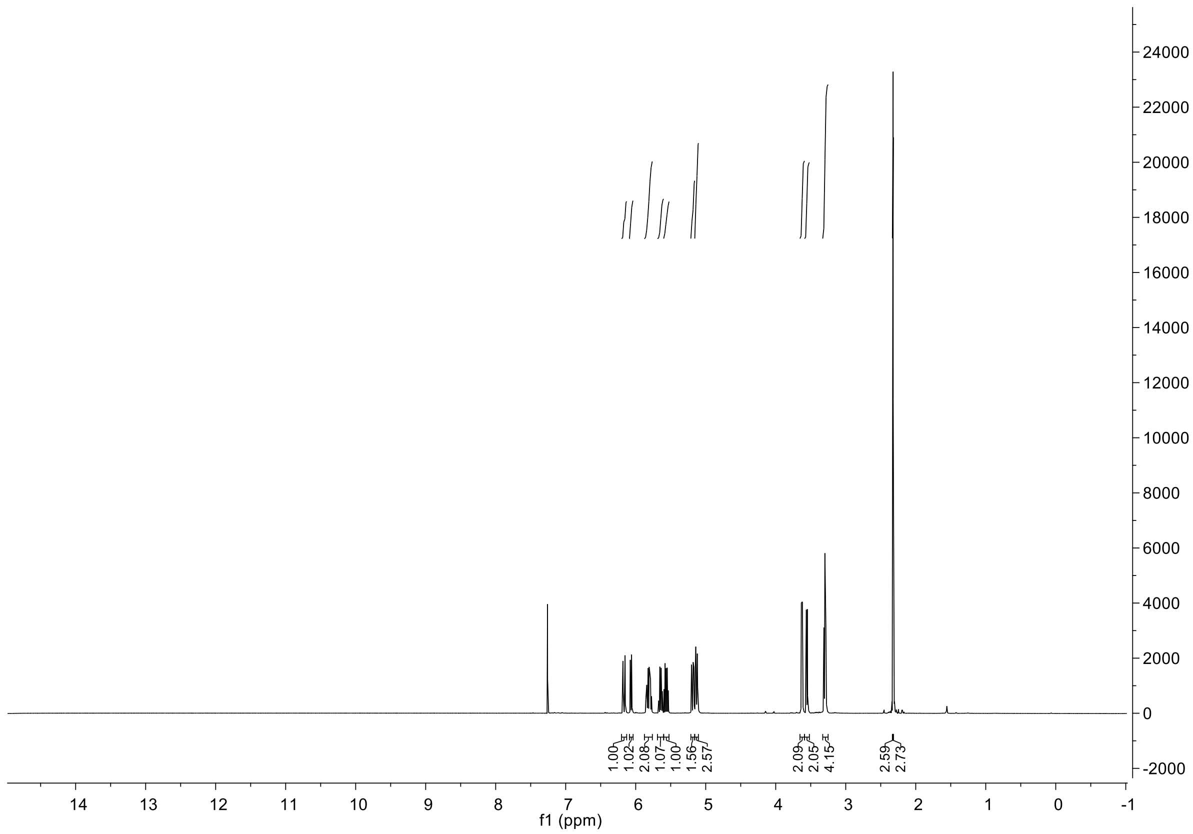
1.00
1.02
2.08
1.07
1.00
1.56
2.57
2.09
2.05
4.15
2.59
2.73
f1 (ppm)

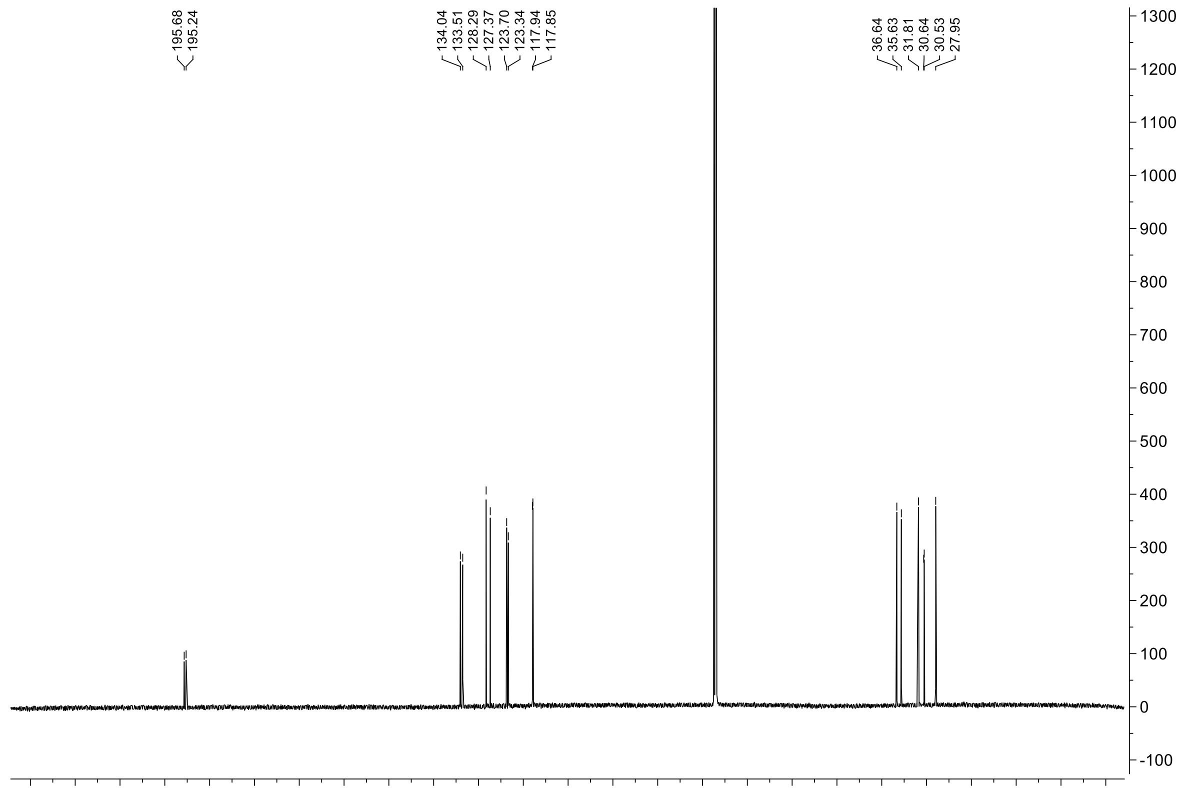
195.68
195.24
134.04
133.51
128.29
127.37
123.70
123.34
117.94
117.85
36.64
35.63
31.81
30.64
30.53
27.95
f1 (ppm)

***S*-(2-(Allylthio)-3-oxopropyl) ethanethioate (18)**

^{1}H NMR (500 MHz, $CDCl_3$): δ = 7.91 (d, J = 10.0 Hz, 1H, C*H*O), 5.75 – 5.66 (m, 1H, C*H*$_2$=CHCH$_2$S), 5.10 – 5.08 (m, 1H, C*H*$_2$=CHCH$_2$S), 5.08 – 5.05 (m, 1H, C*H*$_2$=CHCH$_2$S), 3.89 – 3.79 (m, 1H, SC*H*CHO), 3.02 (ddd, J = 22.1, 13.7, 6.8 Hz, 2H, C*H*$_2$SCOCH$_3$), 2.39 – 2.33 (m, 1H, CH$_2$=CHC*H*$_2$S), 2.31 (s, 3H, C*H*$_3$), 2.26 – 2.19 (m, 1H, CH$_2$=CHC*H*$_2$S) ppm.

^{13}C NMR (126 MHz, $CDCl_3$): δ = 194.9 (S*C*(=O)CH$_3$), 177.8 (*C*HO), 133.7 (CH$_2$=*C*HCH$_2$), 118.4 (*C*H$_2$=CHCH$_2$), 37.7 (CH*C*H$_2$S), 36.7 (S*C*HCHO), 31.5 (*C*H$_2$SCOCH$_3$), 30.6 (*C*H$_3$) ppm.

HRMS (ES) $[M+Na]^+$ calc. 227.0176, found 227.0187 $[C_8H_{12}O_2S_2Na]^+$.

IR (neat): 2924, 1692, 1641, 1439, 1354, 1128, 955, 922, 746, 625 cm^{-1}.

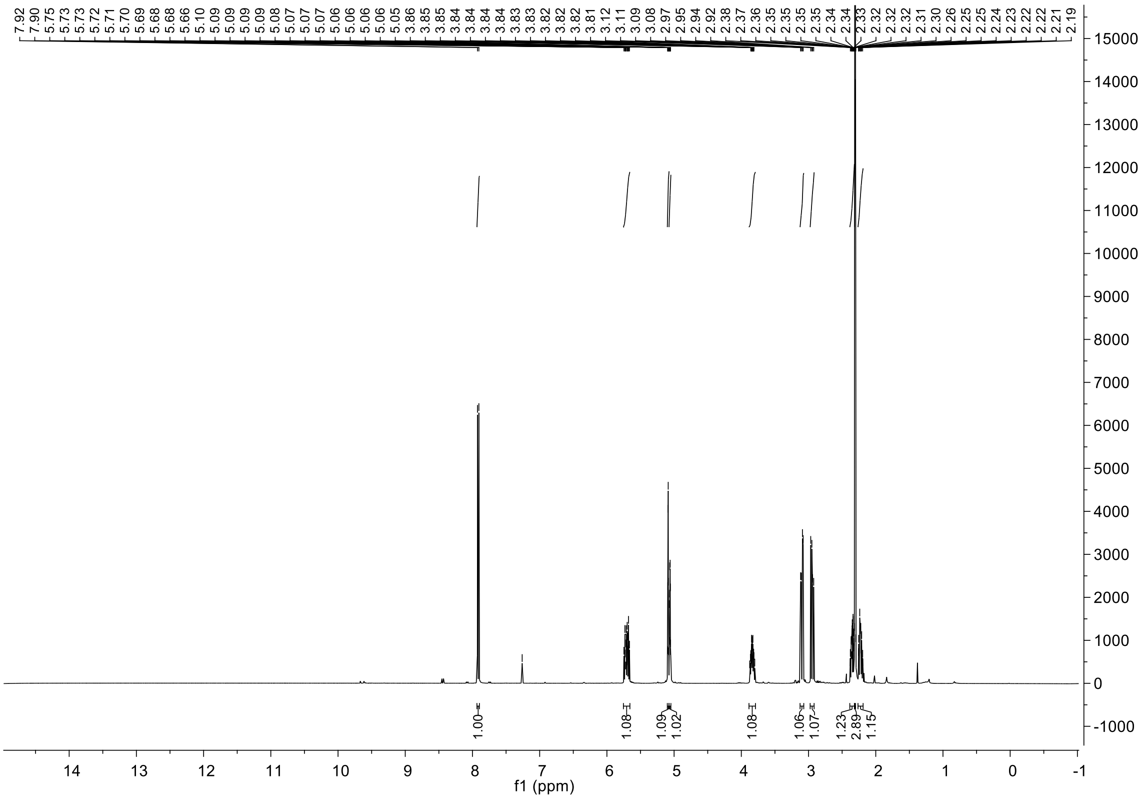

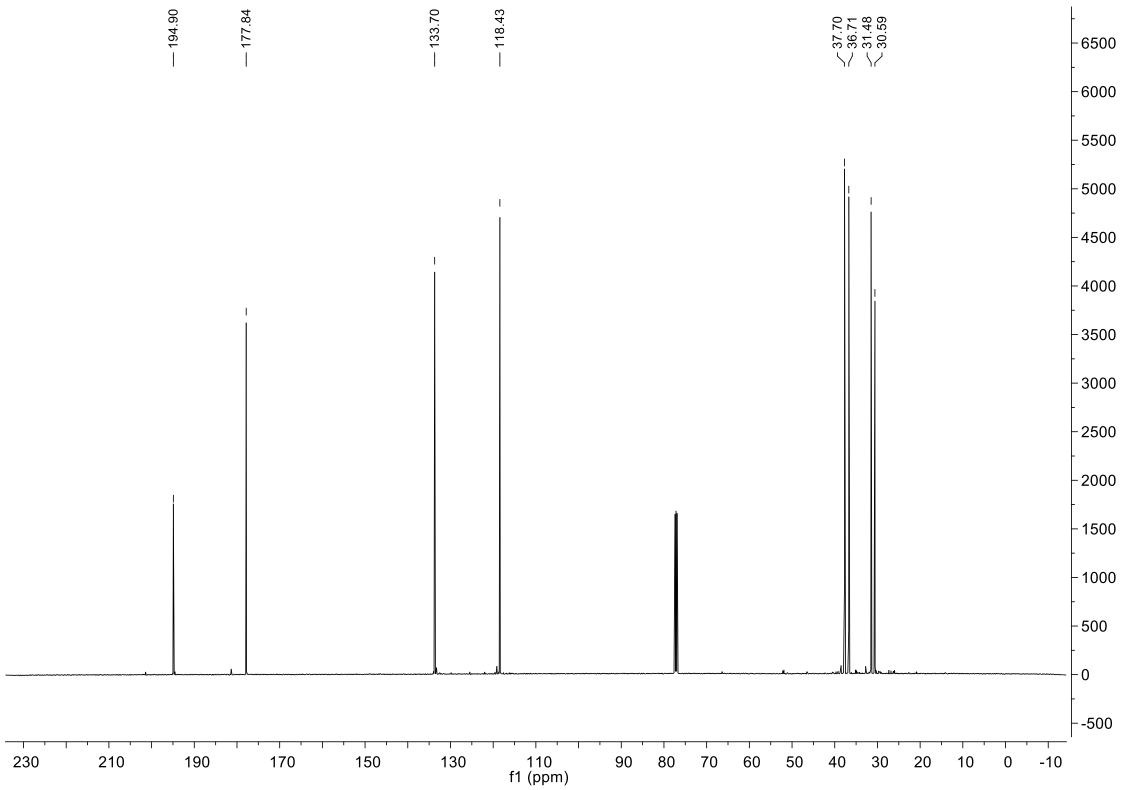
194.90
177.84
133.70
118.43
37.70
36.71
31.48
30.59
f1 (ppm)

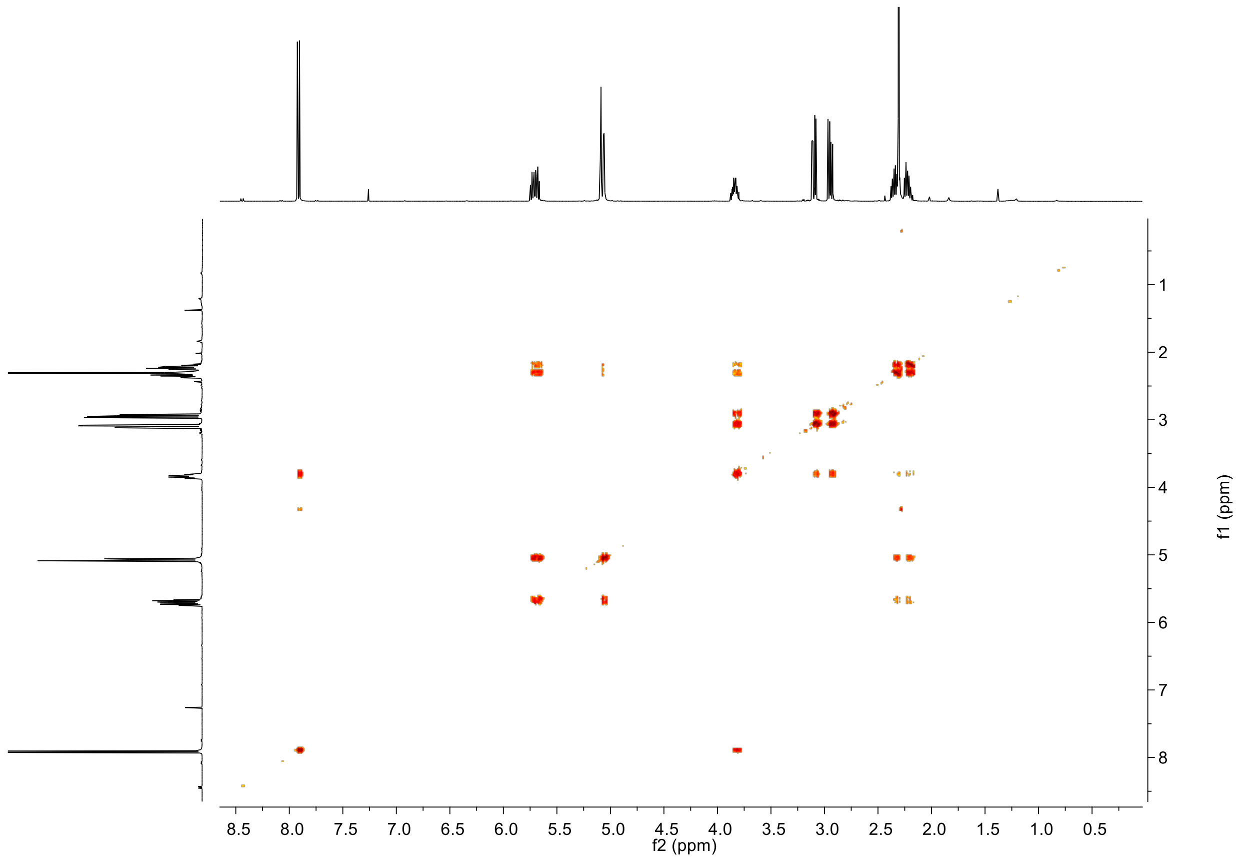
f2 (ppm)
f1 (ppm)

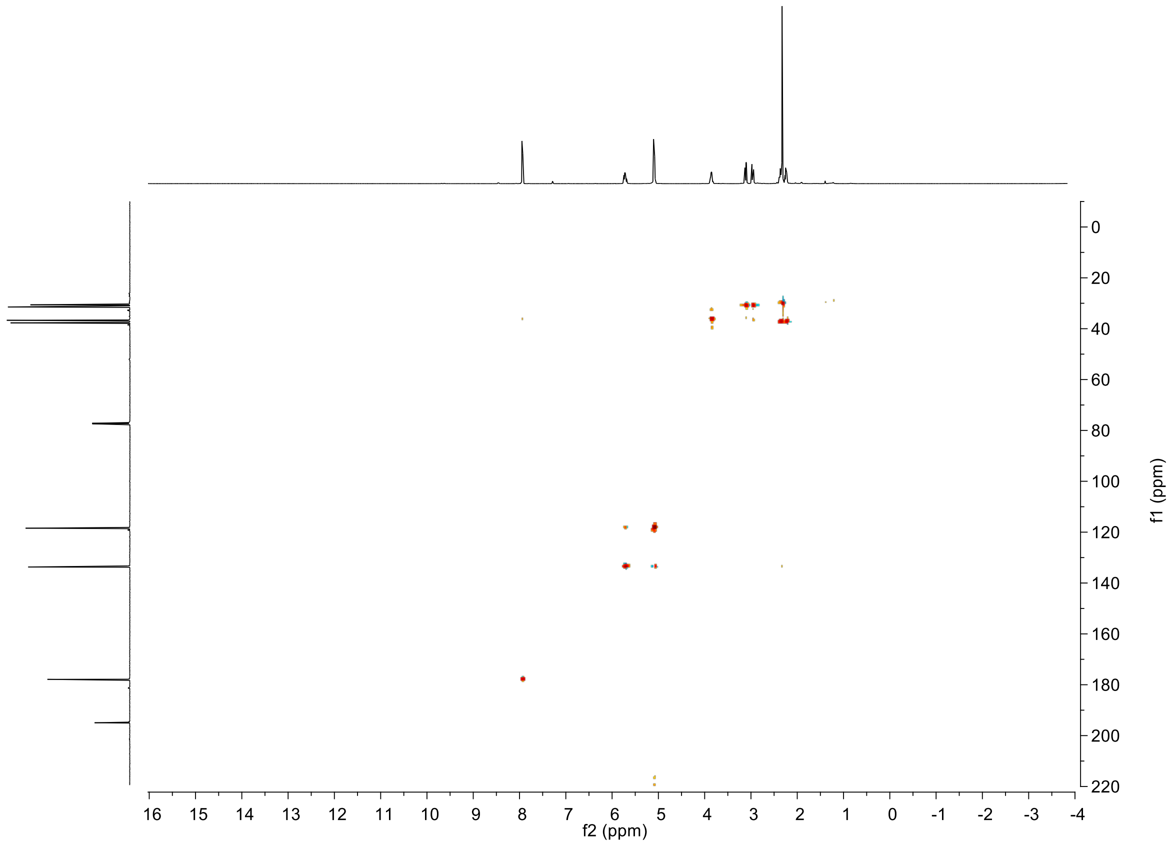

DEPT-135 spectra of 18

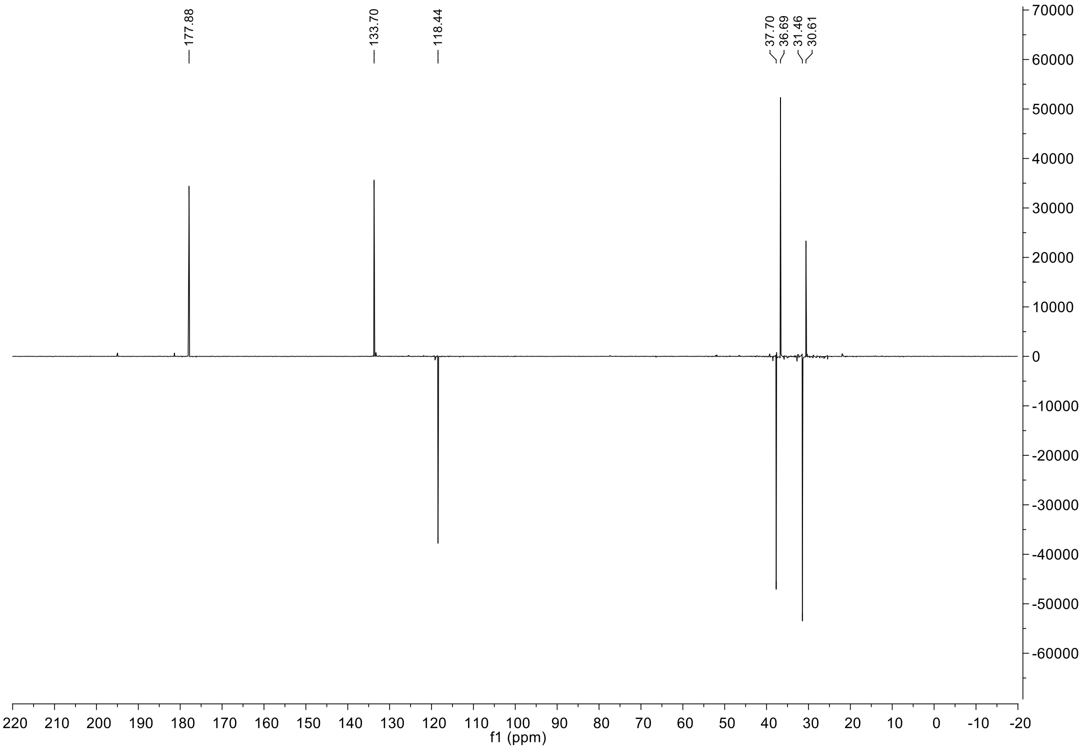

References

[1] G. A. O'Toole, *J. Vis. Exp.* **2011**, *47*, 2437.

[2] T. H. Jakobsen, et al. *Antimicrob. Agents Chemother.* **2012**, *56*, 2314–2325.

[3] A. Nielsen, K. F. Nielsen, D. Free, T, O. Larsen, H. Ingmer, *Antimicrob. Agents Chemother.* **2010**, *54*, 509–512.

[4] J. Castro, A. Moyano, M. A. Pericas, A. Riera, *Synthesis* **1997**, 518–520.

[5] T. Nohara, Y. Fujiwara, T. Ikeda, K. Yamaguchi, H. Manabe, K. Murakami, M. Ono, D. Nakano, J. Kinjo, *Chem. Pharm. Bull.* **2014**, *62*, 477–482.

[6] E. Block, S. Ahmad, M. K. Jain, R. W. Crecely, R. Apitz-Castro, M. R. Cruz, *J. Am. Chem. Soc.* **1984**, *106*, 8295–8296.
